# Genetic Architecture of Capitate Glandular Trichome Density in Florets of Domesticated Sunflower (*Helianthus annuus* L.)

**DOI:** 10.3389/fpls.2017.02227

**Published:** 2018-01-09

**Authors:** Qing-Ming Gao, Nolan C. Kane, Brent S. Hulke, Stephan Reinert, Cloe S. Pogoda, Silas Tittes, Jarrad R. Prasifka

**Affiliations:** ^1^USDA-ARS Red River Valley Agricultural Research Center, Fargo, ND, United States; ^2^Ecology and Evolutionary Biology Department, University of Colorado, Boulder, CO, United States

**Keywords:** sesquiterpenes, capitate glandular trichome, glandular trichome, sunflower, *Helianthus annuus* L., heat shock transcription factor, WRKY transcription factor

## Abstract

Capitate glandular trichomes (CGT), one type of glandular trichomes, are most common in Asteraceae species. CGT can produce various secondary metabolites such as sesquiterpene lactones (STLs) and provide durable resistance to insect pests. In sunflower, CGT-based host resistance is effective to combat the specialist pest, sunflower moth. However, the genetic basis of CGT density is not well understood in sunflower. In this study, we identified two major QTL controlling CGT density in sunflower florets by using a F_4_ mapping population derived from the cross HA 300 × RHA 464 with a genetic linkage map constructed from genotyping-by-sequencing data and composed of 2121 SNP markers. One major QTL is located on chromosome 5, which explained 11.61% of the observed phenotypic variation, and the second QTL is located on chromosome 6, which explained 14.06% of the observed phenotypic variation. The QTL effects and the association between CGT density and QTL support interval were confirmed in a validation population which included 39 sunflower inbred lines with diverse genetic backgrounds. We also identified two strong candidate genes in the QTL support intervals, and the functions of their orthologs in other plant species suggested their potential roles in regulating capitate glandular trichome density in sunflower. Our results provide valuable information to sunflower breeding community for developing host resistance to sunflower insect pests.

## Introduction

Plant trichomes, the hair-like structures on above-ground plant surfaces, are key features governing interaction with the environment, including biotic and abiotic factors. Plant trichomes vary greatly in morphology and function, with at least 300 known types of plant trichomes (Wagner, 1991; Spring, 2000; Werker, 2000). Based on their metabolic activity, plant trichomes are classified into two broad groups: glandular trichomes (GTs) and non-glandular trichomes (non-GTs), which co-exist on plant surfaces like leaves, flowers, stems, and bracts (Hare and Elle, 2002; Rautio et al., 2002). A well-studied example of non-GTs is from the model plant *Arabidopsis thaliana*, which possesses trichomes that are unicellular, unbranched or with two to five branches (Oppenheimer et al., 1991; Larkin, 1994; Szymanski et al., 2000). In contrast, GTs are usually multicellular and consist of differentiated basal, stalk, and secretory cells. GTs have been found on ~30% of all vascular plants, particularly in dicots.

Numerous studies have shown plant trichomes are involved in water protection, absorbing UV radiation, and attracting pollinators by releasing specific chemicals (Skaltsa et al., 1994; Moraes et al., 1998; Paré and Tumlinson, 1999; Benz and Martin, 2006; Lusa et al., 2014). However, the most notable function of plant trichomes is their role in plant defense. Non-GTs serve as physical obstacles to create an unfavorable microenvironment for herbivores, parasites, and pathogens. Some non-GTs form spine- or thorn-like structures that are able to injure and trap insects and other pests during their searching and feeding activities (Pillemer and Tingey, 1976; Riddick and Wu, 2011). On the other hand, GTs are metabolic factories and mainly function in chemical defense in plants (Peiffer et al., 2009; Tian et al., 2012). The biochemical pathways and metabolic profiles in GTs have been studied in great detail in plants (Tissier, 2012). GTs can synthesize and release a wide range of chemicals, including flavonoids, terpenes, alkaloids, acyl-sugars, methyl-ketones and surface proteins (Glas et al., 2012). Recent molecular studies confirm, in different plant species, that trichome development is under the control of a conserved regulatory network, and have identified the function of several key genes in the network (Serna and Martin, 2006; Yang and Ye, 2012; Pattanaik et al., 2014). Compared with leaf tissues, more than 200 genes involved in secondary metabolism and defense-related genes are differentially expressed in trichomes in *Nicotiana tabacum*, and some of these were only expressed in trichomes (Cui et al., 2011). These secondary metabolites are either effective toxins against the pests or attractors to the natural enemies of the pests (Howe and Jander, 2008; War et al., 2012). In some cases, GTs actively release resinous or sticky chemicals on plant surfaces to limit insect movement and function as a physical defense (Simmons and Gurr, 2005; Glas et al., 2012). Studies on wild species of *Medicago* have shown that erect GTs are effective against stem-, leaf-, and fruit-eating pests (Kitch et al., 1985; Danielson et al., 1990), demonstrating their wide role in defense of many types of tissues.

In Asteraceae, GTs have been the subject of many studies for their ability to synthesize various secondary metabolites of ecological and medical importance (Wang et al., 2009; Chadwick et al., 2013; Lv et al., 2017). GTs on tarweed (*Madia elegans*) entrap insects by releasing sticky chemicals and provide carrion as food resource for predators. This indirect defense efficiently decreases the herbivore's activities and increases plant fitness under natural field conditions (Krimmel and Pearse, 2013). Among all the secondary metabolites produced by GTs, sesquiterpene lactones (STLs) are the most prevalent within Asteraceae species. Chemically, STLs derive from the isoprenoid biosynthetic pathway and contain a basic backbone of 15 carbon atoms (Zidorn, 2008). STLs show great structural variety in the arrangement of the basic skeleton and in the composition of the side chain. More than 7,000 distinct molecules of STL have been reported so far (Fraga, 2005). Besides of their ecological role in nature, STLs provide benefits to human health (Chadwick et al., 2013). Artemisinin, a unique STL molecule isolated from sweet wormwood (Artemisia annua), is the most effective treatment for malaria (Covello et al., 2007). Other studies also showed STLs have anti-carcinogenic and anti-inflammatory effects (Li-Weber et al., 2002; Ghantous et al., 2010; Nasim et al., 2011). Consequently, there is great interest in understanding the anatomy, development, biochemistry and molecular biology of GTs in Asteraceae, and the biosynthetic pathways of artemisinin and other STLs have been well studied (Spring and Bienert, 1987; Covello et al., 2007; Zidorn, 2008; Wang et al., 2009; Duarte and Empinotti, 2012; Menin et al., 2012; Michalska et al., 2013; Eljounaidi et al., 2014; Aschenbrenner et al., 2016; Bombo et al., 2016; Lv et al., 2017).

In sunflower (*Helianthus annuus*), there are three main types of trichomes: non-GTs, linear glandular trichomes (LGTs), and capitate glandular trichomes (CGTs). As early as 60–70 h post-germination, these three types of trichomes form on the primordia of the first true leaves, and LGTs and CGTs begin active biosynthesis and secretion of secondary metabolites such as flavonoids, terpenoids, and sesquiterpenes at 72–96 h post-germination (Aschenbrenner et al., 2014). The LGTs are often found among non-GTs and are present on the most parts of sunflower plant, while CGTs only occur on the leaf surfaces and the anther appendages (distal ends) of sunflower florets (Aschenbrenner et al., 2013). In fact, CGTs are the only type of trichome found on anther appendages, where several important sunflower pests feed on pollen and florets, especially the most damaging insect pest of North American sunflower, the sunflower moth (*Homoeosoma electellum* Hulst.). As seen in other Asteraceae, sunflower CGTs synthesize STLs as the primary chemical defense component against herbivores (Göpfert et al., 2005; Rowe et al., 2012).

Since STL are phytotoxic, they are mainly synthesized in the stalk cells of CGTs and secreted and accumulated in the cuticle globes (Göpfert et al., 2005; Amrehn et al., 2013, 2015). Three sesquiterpene synthases (*HaGAS1, HaGAS2*, and *HaTPS2*) and one monooxynase (*HaGAO*) involved in the STL biosynthesis pathway have been identified in sunflower plants (Göpfert et al., 2009; Amrehn et al., 2015). The expression pattern of these genes and the STL biosynthesis activity accompanied anther development, and STL levels in CGTs reached a peak before disk flower opening and remained more or less constant for several days (Göpfert et al., 2009; Amrehn et al., 2015). While feeding or visiting, the insects cause the rupture of the CGTs on anther florets and lead to direct contact with STL. *In vitro* studies have shown that both polar and non-polar extracts of CGTs caused high mortality and retarded growth for the larvae of sunflower moth, and the purified STL species also showed similar effects (Rossiter et al., 1986; Rogers et al., 1987; Prasifka, 2015; Prasifka et al., 2015). Interestingly, the extracts of CGTs are also effective against other floret-feeding insects like yellowstriped armyworm (*Spodoptera ornithogalli*) and migratory grasshopper (*Melanoplus sanguinipes*), indicating a broader spectrum of CGT-mediated defense (Mabry et al., 1977; Isman, 1985).

Three observations have lead us to perform this study: (1) the number of plant trichomes is heritable. In plant taxonomy, trichomes are used as a grouping character since it is a heritable trait across different plant species (Spring, 2000; Reis et al., 2002). Studies in other crop plants have also shown GT density is a quantitatively-inherited trait (Kitch et al., 1985; Agren and Schemske, 1992; Maliepaard et al., 1995; Andrade et al., 2017). However, such information is not available in sunflower. (2) Studies in sunflower have suggested the possibility of developing inbreds or hybrids with high CGT density. Previously, Prasifka (2015) has shown that public sunflower inbred lines (both male and female) present a large range in mean CGT number (>50-fold differences), but wild sunflower and commercial hybrids showed much less variation in the mean of CGT number (~5-fold differences). Thus, breeding a sunflower line with high CGT density is plausible. (3) A positive correlation between CGT number and STL levels in sunflower florets has been documented (Prasifka et al., 2015). Increasing CGT density in sunflower florets could be a strategy for reducing the fitness of insect pests like sunflower moth, thereby reducing damage from insect pests. Therefore, the objective of this study is to identify the QTL responsible for CGT density of anther florets in sunflower. We believe this study will advance our understanding of CGT-mediated resistance to insect pests in sunflower and also benefit sunflower breeding programs by providing the first marker-assisted resource for insect resistance.

## Materials and methods

### Plant materials

Plants of *H. annuus* genotype HA 300 (PI 552938) and RHA 464 (PI 655015; Hulke et al., 2010) were grown in the one-gallon plastic pots under greenhouse conditions with a photoperiod of 16-h light and 8-h dark. High CGT number per floret was observed in maintainer inbred HA300, and low CGT number per floret was observed in restorer inbred RHA 464 (Prasifka, 2015). To map the genetic factors which control CGT number per floret in sunflower, crosses were made between the parents HA 300 (female) and RHA 464 (male) in the 2013 winter greenhouse, and F_1_ plants were grown in a field environment near Fargo, ND, in the summer of 2013. The 300 selfed F_2_ plants and 280 F_3_ plants were grown in the 2014 winter greenhouse and 2014 Fargo field, respectively. A total of 239 F_4_ plants were grown in the 2015 winter greenhouse in the conditions described above and subjected to CGT counting and DNA sampling.

To validate the QTL mapping results of the F_4_ population, a separate diversity panel of 39 inbred lines was selected, which provided a distinct genetic background based on breeding history and pedigree information. These lines were grown in 2016 under greenhouse conditions for CGT counting.

CGTs were sampled and counted in single environments because of the very high heritability of the trait and high cost of evaluation. Prasifka (2015) assessed CGT number from public inbred lines and sampled three replicate plants with three subsamples (florets) from each plant in field environments in 2012 and 2013, finding a very high correlation between the two years (environments; *R*^2^ = 0.98). Similarly, Spring and Bienert (1987) found that while compounds within glands on leaves were affected by lighting, the number of glands per unit area were not. These published data suggest this trait does not possess genotype-by-environment variation generally typical of quantitative traits.

### CGT imaging and counting

Unopened sunflower florets from 239 F_4_ plants were removed with forceps from the outermost 1 cm of the capitulum 1 day before anthesis. After storage at −20°C, one floret per plant was dissected by removing the corolla and making a latitudinal cut through the fused anther tube, after which the unfurled anther tube was attached to an aluminum mount with double-sided carbon adhesive tape (Ted Pella Inc., Redding CA, USA). Mounted groups of 15 florets were sputter-coated with a conductive layer of gold/palladium (Balzers SCD 030, Balzers Union Ltd., Liechtenstein). Scanning electron micrographs (SEM) were obtained using a JEOL JSM-6490LV scanning electron microscope (JEOL USA, Inc., Peabody MA, USA) operating at an accelerating voltage of 15 kV and a magnification of 45 ×. Since the floret area of HA 300 (mean = 14.5, *SE* = 0.75, *n* = 25) and RHA 464 (mean = 13.3, *SE* = 0.2, *n* = 25) are similar, micrographs subsequently were used to quantify the total number of CGT per floret. To estimate the counting error between florets on the same plant (natural variation or trichome loss due to handling), a second floret of each of 30 plants in the sampled F_4_ population was analyzed by SEM and compared to the initial result.

### DNA sampling and sequencing

Leaf tissues were collected from each F_4_ plant, and genomic DNA (gDNA) was extracted from lyophilized leaf material according to the Qiagen DNeasy 96 Plant Kit protocol. Subsequently, library prep was performed using a modified Genotyping by Sequencing (GBS) protocol (Meyer and Kircher, 2010). This protocol used restriction enzymes *Mse1* and *EcoRI* to first digest and fragment the gDNA. The result was a pool of fragments with sticky-ends from restriction cut sites that provide the template for adaptor ligation. Illumina adaptors and barcodes were ligated to the digested fragments. A subset of fragments were then amplified using Illumina PCR primers. The samples were normalized and pooled following PCR, using SequalPrep normalization kit (Thermo Fisher Scientific, USA). Next, the pooled, normalized samples were run out on a 2.5% agarose gel. Using a 1,000 bp ladder, we selected 300–400 bp fragments by cutting out this segment from the gel. The gel segment was then purified using Qiagen gel purification kit according to accompanying protocol. Samples were sent to the University of Texas, Austin, for sequencing on an Illumina HiSeq 2500 sequencer. The results were multiplexed 150 bp single-end barcoded GBS reads.

For HA 300, RHA 464, and a portion of the validation panel, genomic DNA was extracted from lyophilized leaf, and whole genome sequencing was performed. Genomic libraries were prepared using Nextera® XT DNA library prep kits (Illumina®) according to the protocol. Each gDNA sample was diluted to the appropriate concentration using a Qubit 3.0 fluorometer (Thermo Fisher Scientific, USA). Each sample was barcoded by the unique dual index adapters Nextera® i5 and i7. Resulting libraries were cleaned using solid-phase reversible immobilization (SPRI) to remove fragment sizes less than 300 base pairs via an epMotion 5075TMX automated liquid handling system (Eppendorf North America). Sample quality control (QC) was conducted to ensure appropriate sample concentration and fragment size using a Qubit 3.0 fluorometer and an Agilent 2100 Bioanalyzer prior to normalizing the loading concentration to 1.8–2.1 pM with 1% PhiX control v3 added (Illumina®). Samples that passed QC were processed for paired-end 150 base pair reads on the Illumina NextSeq® sequencer. Whole genome sequencing was conducted at the BioFrontiers Institute Next-Generation Sequencing Facility at University of Colorado. The remainder of the validation panel was sequenced as part of the sunflower SAM population by the Genome Quebec Innovation Centre at McGill University, Montreal, QC, Canada (Mandel et al., 2013; Burke, pers. comm.).

### SNP marker calling

All GBS reads were demultiplexed using process_radtags (v1.45) command from the Stacks software suite (Catchen et al., 2013). Default parameters were used with the exception of adding the “r” and “disable_rad_check” flags. After demultiplexing, each sample was checked for overrepresented sequences with FastQC (v0.11.5; Andrews, 2010). Trimmomatic (v0.35; Bolger et al., 2014) was used with default parameters to trim and clean the demultiplexed sequences. Reads with overrepresented sequences as identified by FastQC were also trimmed by appending them to the “TruSeq3-SE.fa” adapters file distributed with Trimmomatic. Reads were aligned to the sunflower reference genome (HA 412HO_v1.1) using the BWA mem (v0.7.12-r1039; Li and Durbin, 2009) algorithm with default parameters and the duplicates in alignments were marked with Picard (v2.8.1; Broad Institute). A realignment and base call recalibration were performed with GATK (Genome Analysis Toolkit) and followed the GATK Best Practices. Finally, the alignment quality was checked with Qualimap (v2.2.1; Okonechnikov et al., 2015), which rendered the data ready for variant calling. The variant calling was performed with freebayes (v1.1.0-1-gf15e66e; Garrison and Marth, 2012), and WGS data from the parental lines (HA 300 and RHA 464) were jointly genotyped to improve the variant calling quality. The variant sites were filtered using vcftools (v0.1.13; Danecek et al., 2011). We kept only bi-allelic single nucleotide polymorphic (SNP) sites, and required a minimum site quality of 30 and a minor allele frequency greater than 0.05.

In addition to filtering sites with vcftools, we applied a protocol to filter sites that are likely to be misassembled multi-copy portions of the genome. Repetitive sites are susceptible to misassembly with respect to the reference sequence and may result in several reads erroneously mapping to the same locus. To reduce the number of these sites in our variant calls, we assessed sequencing depth of whole genome shotgun sequence reads from four modern sunflower cultivars and one landrace (Table S1) aligned to the reference genome. We used the samtools depth command to produce the number of mapped sequences per site for each individual. We summed the depths at each position, and calculated the frequency of summed depth values across samples. We then plotted the summed depth values against their frequencies, providing a visual means to choose a range of summed depth values that are most likely to correspond to well-assembled single copy sites of the genome. We then filtered our vcf table to only include the identified single-copy sites. Out of 3,200,466 diagnostic sites differing between the parental genomes, we identified 1,138,669 single-copy diagnostic SNPs. After filtering the vcf table, missing genotypic information from the GBS data of the F_4_ population was trio imputed using our own custom phaser2.pl and genotyper7.pl software (Kane, 2017), and leveraging the whole genome sequence of the parent lines HA 300 and RHA 464. These programs are designed to accurately impute for high-quality single-copy, diagnostic SNPs. Because of our extensive filtering, and high quality data, these assumptions were met.

### QTL analysis

QTL analyses were carried out using the R/qtl package in R version 3.2.3 (Broman et al., 2003). The genetic map was constructed with the est.map function (assuming a genotyping error rate of 0.001). Single QTL analysis and LOD scores were calculated by a single QTL genome scan (scanone function) with standard interval mapping (0.1 cM steps, assuming a genotyping error rate of 0.001). Pairwise QTL interactions were calculated using a two-dimensional QTL scan (scantwo function) via standard interval mapping (0.1 cM steps, assuming a genotyping error rate of 0.001). LOD significance thresholds for type I error rates of α < 0.05 were determined by running 1,000 permutations on the single- and two-dimensional QTL scan. In addition, the stepwiseqtl function (max.qtl = 4, additive.only = FALSE) was used to perform forward/backward stepwise search, and find the QTL model which has the highest LOD score. The fitqtl function was used to create a QTL project to fit the phenotypic data with the selected model, and one QTL was omitted at a time to obtain an ANOVA table.

### Validation of the mapped QTL

To validate the QTL results, the SNP markers were called from the validation population together with HA 300 and RHA 464, as described above. The SNP markers derived from the QTL support interval were extracted. Monomorphic SNPs between the two parent lines were removed, and the heterozygous SNPs and markers with missing data in one of the parent lines were also filtered out. The SNP markers were treated as fixed effects and all possible marker pairs were tested by fitting a regression function in R as y ~ SNP_*m*_ + SNP_*n*_, where y is the CGT number, SNP_*m*_ is the effect of the *m*th marker from chromosome 5, and SNP_*n*_ is the effect of the *n*th marker from chromosome 6. The significance threshold for multiple comparisons was determined by the adjusted *p*-values with the false discovery rate at 0.05. (Benjamini and Yekutieli, 2001).

### Identification and phylogenetic analysis of candidate genes within the QTL support interval

To identify the candidate genes with the highest likelihood of influence on the phenotype, we first manually checked through the QTL support interval region in the sunflower reference genome (INRA Sunflower Bioinformatics Resources, 2014) and listed all the annotated genes (Table S5). We then filtered the list based on two criteria: (1) significant association between a co-located SNP and CGT number in the validation population; and (2) putative gene function. The sequences of selected candidate genes were further characterized by two approaches, NCBI (National Center for Biotechnology Information) ORFfinder search and BLAST homology search. All ORFs within these sequences were identified using NCBI ORFfinder with the following parameters: a minimal ORF length of 150 nucleotides, the standard genetic code, ATG and alternative start codons, and ignore nested ORFs. BLASTp searches were carried out with the given ORFs to obtain the most similar protein sequences in Asteraceae, Solanaceae, Malvaceae and Brassicaceae. The sequences with an E-value less than 10^−5^ were selected, combined, and searched for the presence of conserved protein domains using ScanProsite (Sigrist et al., 2012). Comparisons were made among HA 412HO (reference sequence), HA 300, and RHA 464 for genomic sequence differences, alternative splice sites, and changes in protein domains at the selected loci. For phylogenetic analysis, the conserved protein domain sequences with 10–15 additional amino acids on the N-terminal and C-terminal ends were used for alignment. The alignment was done with the L-INS-i strategy implemented in MAFFT (version 7.310; Katoh, 2002). The BLOSUM62 scoring matrix and a gap opening penalty of 1.5 were selected to assess the phylogenetic relationship among the protein sequences. After the alignment, a neighbor-joining tree was constructed with PhyML including the Smart Model Selection approach (version 3; Guindon et al., 2010; Lefort et al., 2017), bootstrap reassembling with 1,000 bootstrap samples. The phylogenetic tree was colorized according to protein families in Archaeopteryx (version 0.9920 beta; Han and Zmasek, 2009).

## Results

### Capitate glandular trichome density in mapping population

**Figure 1:**
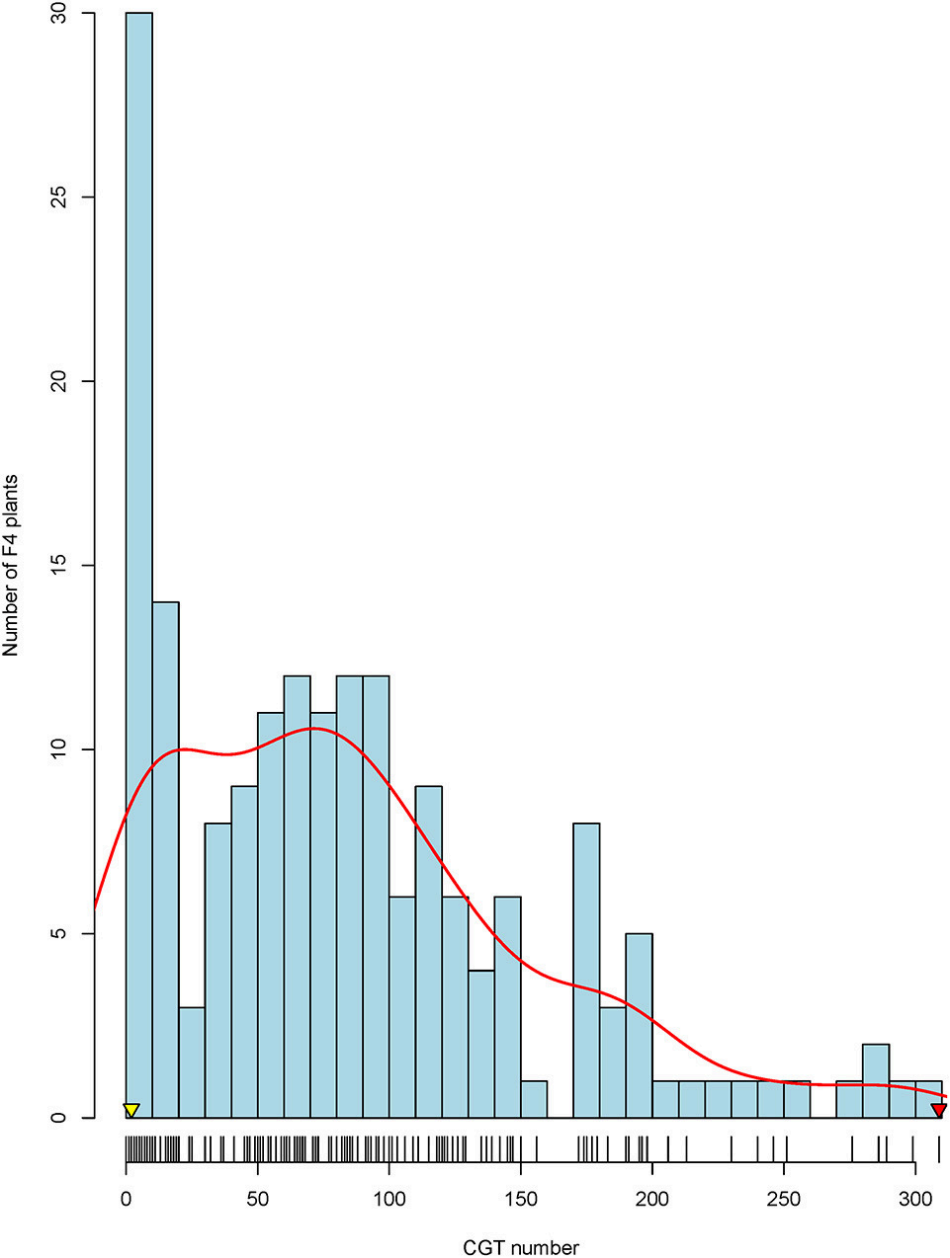
Frequency distribution of capitate glandular trichomes (CGTs) in the F_4_ mapping population. The arrowheads indicate the CGT number of the parental lines, HA 300 (red arrow) and RHA 464 (yellow arrow).

The two parents presented a significant difference in CGT density, with the male parent RHA 464 having ~2 CGT per floret and the female parent HA 300 having ~300 CGT per floret (Figure 1). The CGT numbers per floret were counted for 239 F_4_ plants, and 179 plants with good genotypic data quality were selected as the final mapping population. Based on frequency distribution, 179 F_4_ plants were classified into three groups (Figure 1). Of these 179 F_4_ plants, 25 plants (13.9%) had high CGT density which is more than 150 CGT per floret, 109 plants (60.9%) had medium CGT density which is 25–150 CGT per floret and 45 plants (25.2%) had low CGT density which is less than 25 CGT per floret. Shapiro-Wilk normality test (Shapiro and Wilk, 1965) indicated that the CGT number in the F_4_ population was not normally distributed (W = 0.915, *p* < 1.1 × 10^−8^), and the distribution of CGT number was moderately skewed toward the lower CGT number (Skewness = 0.98). The original mapping population with 239 individuals showed a similar distribution of CGT number (Skewness = 0.97; W = 0.916, *p* < 1.1 × 10^−10^).

To estimate the potential error in CGT counting (natural variation or trichome loss due to handling), 30 plants from the F_4_ population were randomly selected for recounting. A second floret from each of these 30 plants was counted, and the CGT numbers from first floret and second floret were highly correlated (*r* = 0.98, *p* < 2.2 × 10^−16^) (Figure S1).

### Genetic map construction

**Figure 2:**
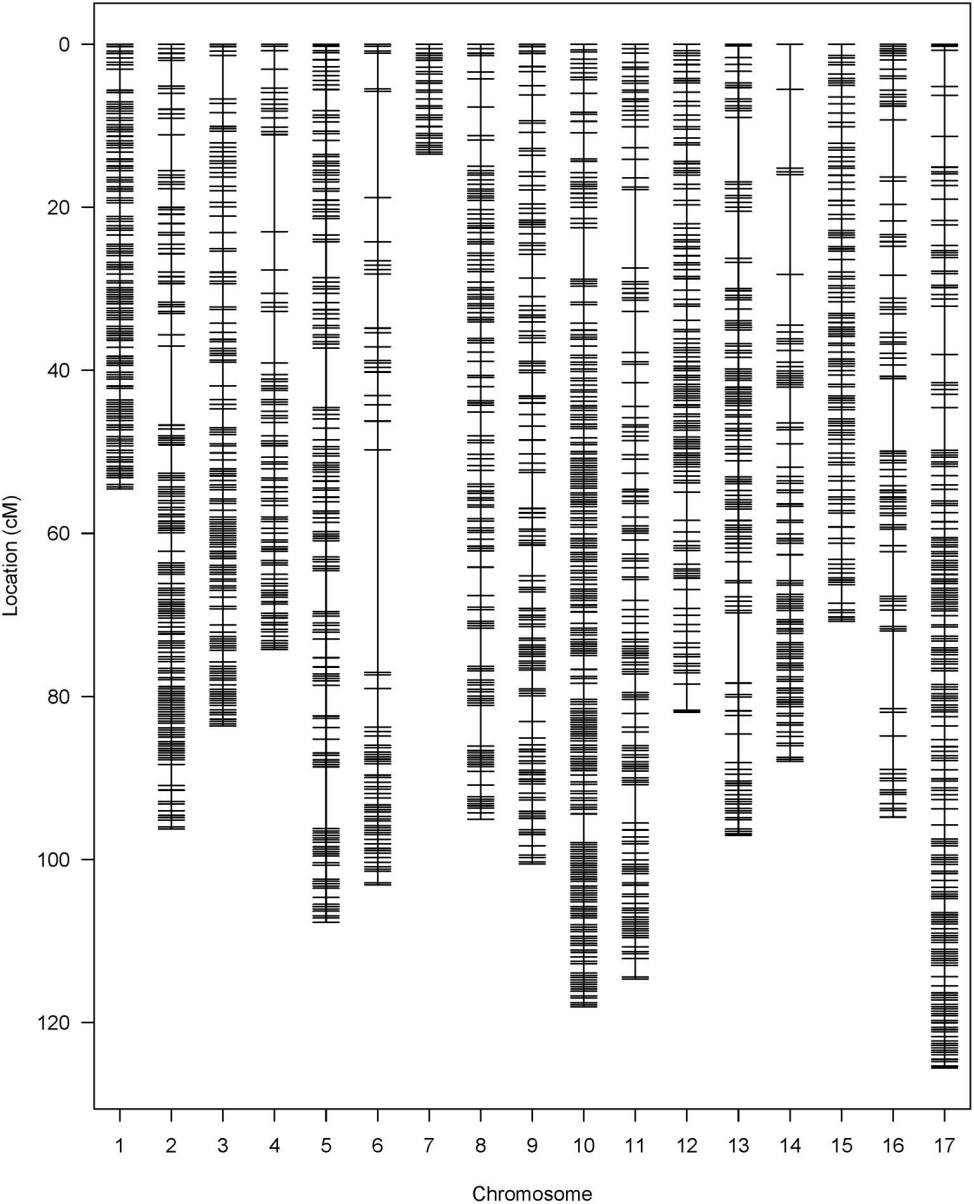
SNP distribution in the HA 300 × RHA 464 genetic linkage map.

A total of 443.2 M raw GBS reads were generated from 239 F_4_ plants with the Illumina HiSeq 2500 sequencing system. After trimming the adapters and overrepresented sequences with Trimmomatic (v0.35; Bolger et al., 2014), the number of remaining reads was 210.1 M. From these 210.1 M reads, 97.9% (205.7 M) of the reads were successfully aligned to the sunflower reference genome HA 412HO_v1.1., and the mean length of reads was 74.3 bp. Before performing variant calling, five individuals were removed due to poor genotyping quality. A total of 16,028,511 SNPs was produced in the initial variant calling, and 1,138,669 single-copy diagnostic SNP were retained after applying several filters (minQ > 30, MAF > 0.05, biallelic only). To further control genotypic data quality, we also checked each individual in the F_4_ population, and the individuals with low coverage on one or more chromosomes (less than 10 SNPs per chromosome) were dropped. A total of 179 individuals with acceptable sequence quality were kept to conduct imputation. A total of 1.09 million SNPs was generated from the imputation, and monomorphic markers and markers with LD above 0.9 were filtered out first, followed by removing SNPs with high missing rate and significant distortion from expected Mendelian ratio. Finally, 2,121 high quality SNPs were kept and used for constructing the genetic linkage map (Figure 2).

The 2121 SNP markers were distributed in all 17 chromosomes, and the number of markers per chromosome varies from 32 on chromosome 7 to 238 on chromosome 10 (Table 1). The total length of the map was 1,519.52 cM, and the size of chromosomes varies from 13.48 cM (chromosome 7) to 125.58 cM (chromosome 17). Chromosome 7 had the fewest SNP markers and shortest map distance. The average density per marker was 0.72 cM, and the largest gap between two adjacent loci was 27.32 cM on chromosome 6.

**Table 1:** Summary of SNP loci on the HA 300 × RHA 464 linkage map.

**Linkage groups**	**Number of SNPs**	**Map length (cM)**	**Density (cM/marker)**	**Largest gap**
1	129	54.56	0.43	2.58
2	149	96.23	0.65	9.71
3	134	83.62	0.63	5.36
4	86	74.24	0.87	11.91
5	147	107.71	0.74	7.58
6	73	103.31	1.43	27.32
7	32	13.48	0.43	0.85
8	130	95.07	0.74	5.01
9	129	100.54	0.79	4.43
10	238	118.06	0.50	6.30
11	141	114.65	0.82	9.63
12	129	81.91	0.64	3.49
13	123	97.05	0.80	8.63
14	95	87.95	0.94	12.24
15	112	70.77	0.64	2.29
16	86	94.79	1.12	9.51
17	188	125.58	0.67	5.94
Total	2121	1519.52	0.72	/

### QTL analysis

**Figure 3:**
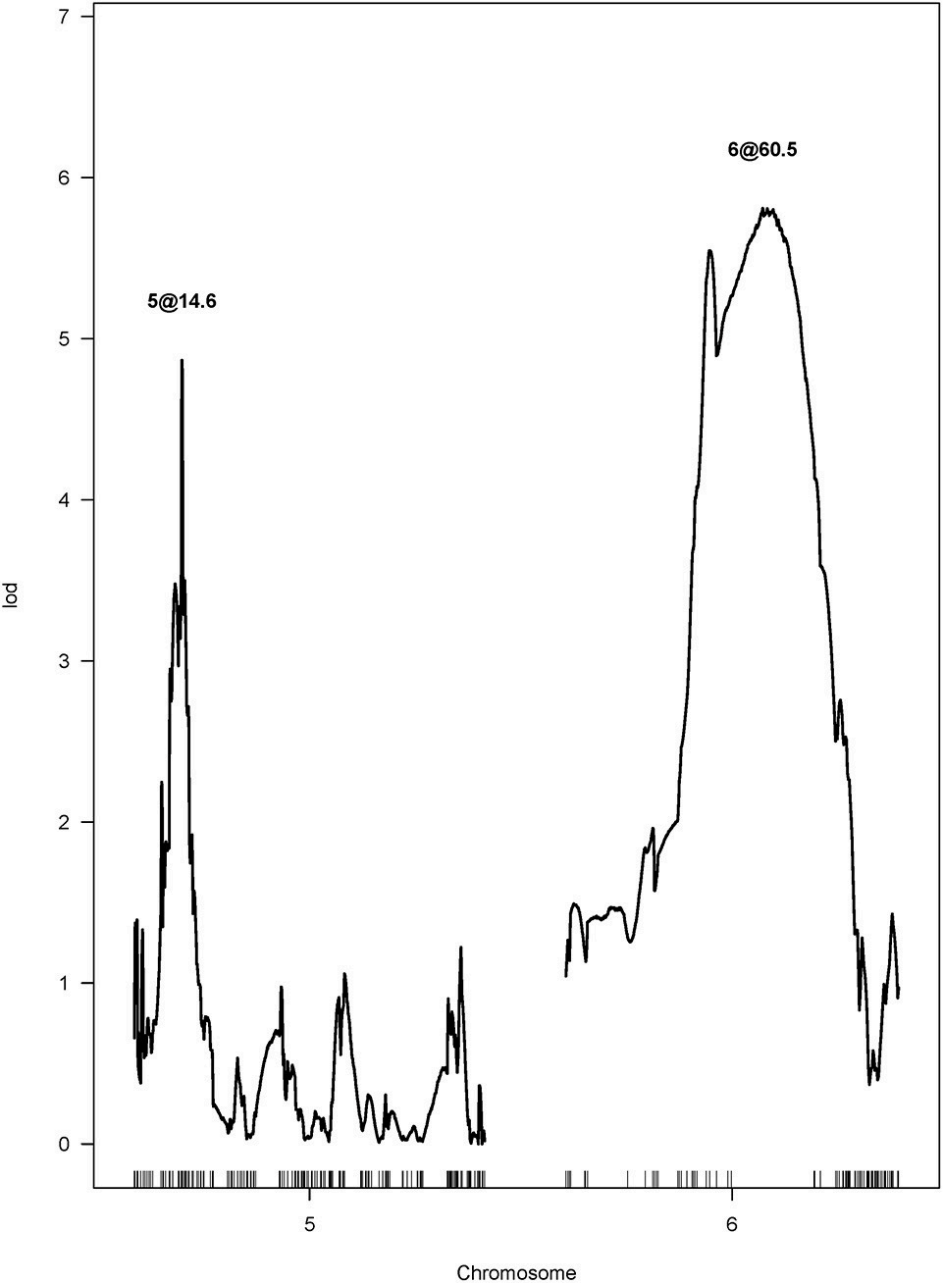
LOD curves for the capitate glandular trichome (CGT) phenotype in the F_4_ mapping population, calculated by two-QTL scan.

After performing a single QTL scan on the data collected from the F_4_ population, two putative QTL were revealed. One was mapped to chromosome Ha5 at the 14.64 cM position, and the other one was mapped to chromosome Ha6 at the 60.72 cM position (Figure 3). Both QTL had LOD scores greater than the LOD threshold (4.70, α = 0.05), which was 95 percentile of the distribution of genome-wide max LOD obtained by 1,000 permutations (Table S2). Therefore, a two-QTL model scan was performed with the scantwo function, and the same pair of QTL on Ha5 and Ha6 were identified (Table S3). In this two-QTL model, the additive effect of these two QTL was strongly supported by the data, but no epistasis interaction was detected. The two-QTL additive model (Ha5@14.6, Ha6@60.5) explained 24.58% of CGT number variation for the F_4_ mapping population, while the single QTL model explained 11.61 and 14.06% of the CGT number variation, respectively (Table 2). To confirm the QTL model, we also performed a forward/backward stepwise search, allowing four QTL at maximum, and found the two-QTL model had the best LOD score. The closest SNPs to each QTL were identified, and the phenotypes were plotted against genotypes at the putative QTL (Figure S2, Table S4). The 1.5—LOD support interval for SNP marker Ha5_11356218 was 12.1–15.7 cM, which extended from 8.07 to 12.65 Mbp on the physical map, and the support interval of SNP marker Ha6_8364901 was 41.3–76.3 cM, which covered from 7.42 to 8.58 Mbp on the physical map (Table 2, Figure S3).

**Table 2:** Two QTL for capitate glandular trichome (CGT) density identified in the HA 300 × RHA 464 mapping population.

**QTL**	**Closest SNP**	**LOD**	**Explained variance (%)**	***p*-value**	**1.5-LOD support interval**
Ha5@14.6	Ha5_11356218	10.06	11.61	1.85 × 10^−5^^***^	12.1–15.7 cM
Ha6@60.5	Ha6_8364901	12.19	14.06	2.18 × 10^−6^^***^	41.3–76.3 cM
Ha5@14.6 + Ha6@60.5^a^	/	10.97	24.58	4.89 × 10^−10^^***^	/
Ha5@14.6^*^Ha6@60.5^b^	/	1.62	0.935	0.45	/

*** *Significant at p < 0.001 as determined by permutation testing*.

a *Additive effect of two QTL*.

b *Interaction effect of two QTL*.

### Validation of the mapped QTL

To validate the QTL analysis, we selected 39 inbred lines which were whole genome sequenced with 10 × coverage, and had phenotypic data from a previous study (Prasifka, 2015). With the WGS reads, 609914 SNPs were produced from variant calling and 485516 good quality SNPs were kept after applying the additional filters (minQ > 30, MAF > 0.05, biallelic only, max missing rate <10%). A phylogenetic tree was constructed from these 485516 SNPs using the SNPhylo pipeline (Lee et al., 2014; Figure S4). As shown in Figure S4, the two parent lines, RHA 464 and HA 300, were clustered into two distinct subgroups, and the other 37 lines in the validation population presented additional genetic backgrounds.

**Figure 4:**
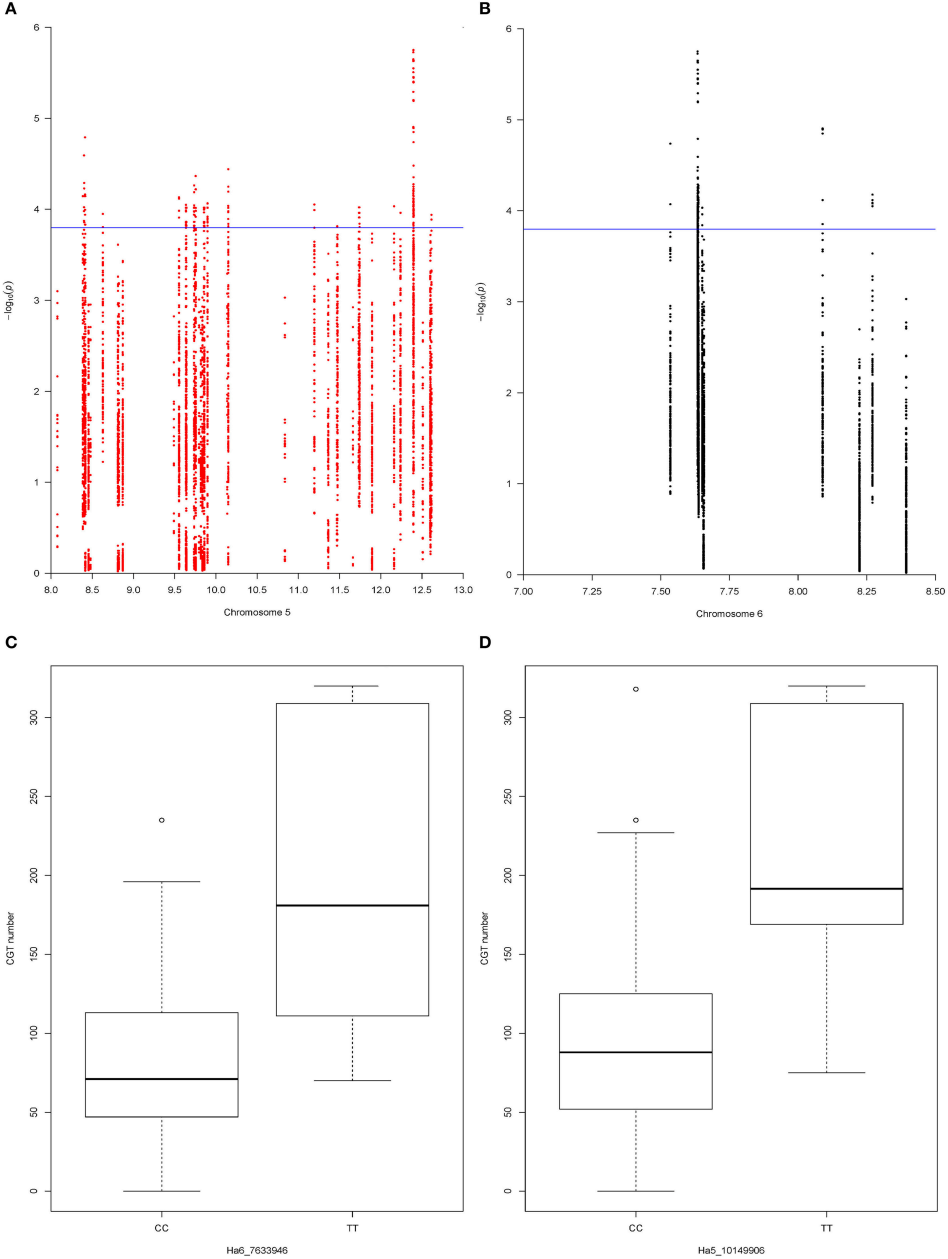
**(A)** The −log ^p*-value*^ for each SNP marker in the chromosome 5 QTL support interval. Values were plotted against their respective physical positions (Mbp). The blue line represents the significance threshold (*p* = 1.6 × 10^−4^). **(B)** The −log ^*p-value*^ for each SNP marker in the chromosome 6 QTL support interval. Values were plotted against their respective physical positions (Mbp). The blue line represents the significance threshold (*p* = 1.6 × 10^−4^). **(C)** Effect of SNP marker Ha6_7633946 on CGT number. The box plot of each allele at SNP marker Ha6_7633946 shows the significant association with CGT number in the validation panel (*p* = 0.0004). The horizontal bar (bold) indicates the median, discontinuous lines represent the upper and lower quartile, and the outlier data are labeled by a circle. **(D)** Effect of SNP marker Ha5_10149906 on CGT number. The box plot of each allele at SNP marker Ha5_10149906 shows the significant association with CGT number in the validation panel (*p* = 0.004). The horizontal bar (bold) indicates the median, discontinuous lines represent the upper and lower quartile, and the outlier data are labeled by a circle.

To test the two-QTL model, which was suggested by the QTL mapping results, we retrieved all the SNP markers from the QTL support intervals. A total of 990 SNP markers were extracted from the QTL support interval on chromosome 5, with a marker density of 0.22 SNP per kb, and 363 SNP markers were extracted from the QTL support interval on chromosome 6, with a marker density of 0.31 SNP per kb. After filtering these SNP markers, as described above, we kept 385 SNP markers from the QTL support interval on chromosome 5 and 33 SNP markers from the QTL support interval on chromosome 6. Next, we tested pairs of SNP markers, one from each QTL, with a linear regression function in R [lm(y ~ SNP_*m*_ + SNP_*n*_), where y is the CGT number, SNP_*m*_ is the effect of the *m*th marker on chromosome 5 and SNP_*n*_ is the effect of the *n*th marker on chromosome 6]. In total, 12705 two-QTL models were tested, and 414 of these models were detected as significant with the adjusted *p* < 0.05 (*p* = 1.6 × 10^−4^) (Figures 4A,B). The best two-QTL model explained 59.9% of total phenotypic variation.

### Identification of putative genes controlling CGT number

To identify putative genes which control CGT number in sunflower, we manually checked through the physical regions, which included the Ha5_11356218 marker support interval and the Ha6_8364901 marker support interval, with sunflower genome JBrowse (INRA Sunflower Bioinformatics Resources, 2014). A total of 105 genes were predicted within the Ha5_11356218 marker support interval on chromosome 5, and 35 genes were predicted within the Ha6_8364901 marker support interval on chromosome 6. A list of these genes with SNP marker data and expression profile in flower tissues is shown in Table S5.

Since some of the SNP markers in the QTL support intervals were within these genes, we found 16 genes on chromosome 5 and five genes on chromosome 6 with variation that had significant associations with CGT number (Figures 4A,B). Based on gene annotations that suggested plausible functional association, the genes co-localized with Ha5_10149906 and Ha6_7633946 were selected for further characterization. This pair of markers had strong association with CGT number (adjusted *p* < 0.05, *p* = 3.6 × 10^−5^) and the two-QTL model explained 47.2% of phenotypic variation in the validation data. The genotypic data at these loci together with phenotypic data are shown in Table 3. A single factor ANOVA was also used to test the association between each SNP marker and CGT number. The marker Ha5_10149906 showed strong association with CGT number (*p* = 0.004), explaining 20.3% of the phenotypic variation, and the single SNP marker Ha6_7633946 also showed significant association with CGT number (*p* = 0.0004), explaining 31.1% of the phenotypic variation (Figures 4C,D).

**Table 3:** Genotypes of two QTL and phenotypic data in the validation population (/ represents missing data).

**Inbred lines**	**CGT number**	**Ha5_10149906**	**Ha6_7633946**
RHA 464 (male parent)	2	CC	CC
HA 300 (female parent)	309	TT	TT
HA 124	28	CC	CC
HA 133	188	TT	TT
HA 207	195	TT	CC
HA 232	92	/	/
HA 248	334	/	/
HA 291	60	CC	CC
HA 292	108	CC	CC
HA 301	30	/	/
HA 305	235	CC	CC
HA 321	174	CC	TT
HA 349	133	CC	CC
HA 371	109	TT	CC
HA 383	75	TT	/
HA 404	318	CC	TT
HA 441	169	TT	CC
HA 445	136	/	/
HA 456	88	CC	CC
HA 467	140	/	/
HA 821	320	TT	TT
HA 851	117	CC	CC
HA 853	111	CC	TT
HA 89	71	CC	CC
ND-NONOIL M3	70	CC	TT
RHA 270	196	CC	CC
RHA 272	49	CC	CC
RHA 273	0	CC	CC
RHA 274	91	CC	CC
RHA 278	227	CC	TT
RHA 331	108	CC	CC
RHA 374	101	CC	TT
RHA 377	45	CC	CC
RHA 386	55	CC	CC
RHA 388	65	CC	CC
RHA 396	0	CC	CC
RHA 400	160	CC	TT
RHA 408	55	CC	CC
RHA 468	118	/	/
RHA 855	13	CC	CC

**Figure 5:**
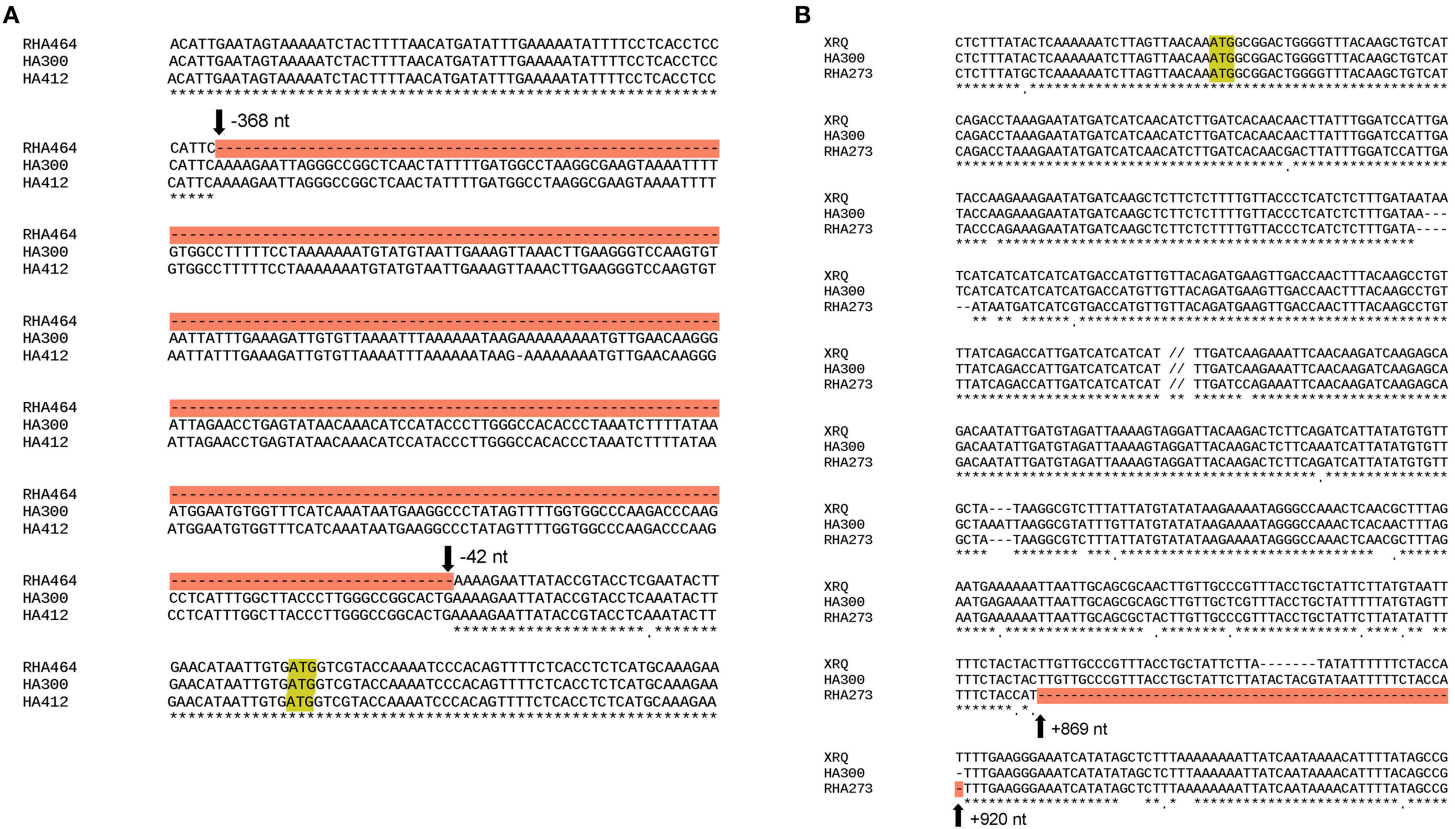
**(A)** Alignment of genomic sequence of the heat shock transcription factor-like (*HSF*) gene from two parental lines and the reference genome HA 412HO. The start codon (ATG) is shaded in yellow, and the deletion in RHA 464 is shaded in red. **(B)** Alignment of genomic sequence of the *WRKY*-like gene from two parental lines and the reference genome XRQ. The start codon (ATG) is shaded in yellow, and the deletion in RHA 273 line is shaded in red.

The SNP marker Ha5_10149906 is located within the second exon of the gene Ha5g003120, which is annotated as a member of heat shock transcription factor (HSF) family (Table S5). The HSF proteins are not only involved in heat stress responses but also participate in many other developmental/physiological activities such as cell division and root growth (Westerheide et al., 2012). As shown in the phylogenetic tree (Figure S5), sunflower Ha5g003120 gene is orthologous to *Arabidopsis thaliana* gene *HSF2A*. Furthermore, we obtained the genomic sequence of gene Ha5g003120 from the two parents, HA 300 and RHA 464, and the reference genome HA 412HO, and performed alignments. Interestingly, the genomic sequence in RHA 464 has a 326 bp deletion in the promoter region of Ha5g003120, but this deletion is not present in HA 300 and HA 412HO (Figure 5A).

**Figure 6:**
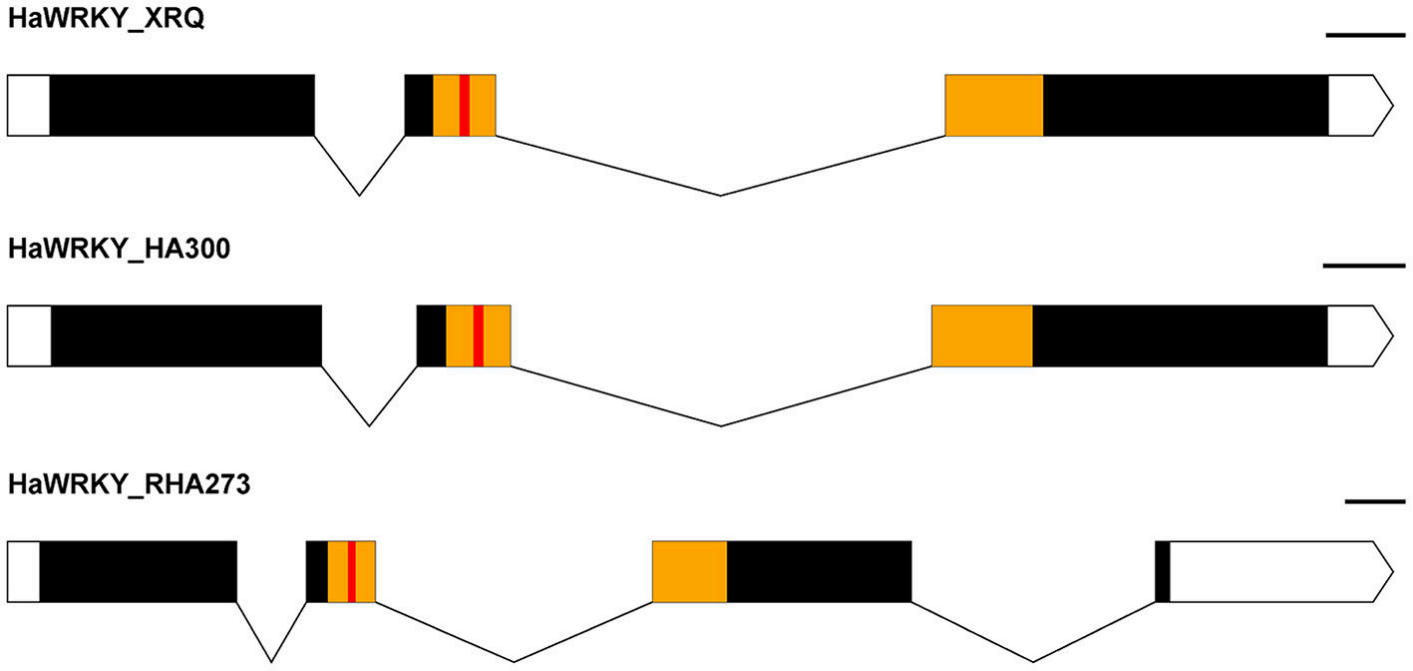
Exon-Intron map of a *WRKY*-like sequence from the parental lines (HA 300, RHA 273) and reference genome XRQ, which shows a change in the splicing pattern in RHA 273 due to a deletion. White boxes represent the UTRs, solid lines represent the introns, black boxes represent the exons, yellow boxes represent the conserved *WRKY* domain, and the red boxes indicate the *WRKY* motif. The scale is 100 bp.

The SNP marker Ha6_7633946 is located in the 3′-UTR (untranslated region) of the gene Ha6g003560, which is annotated as a member of the WRKY transcription factor family (Table S5). WRKY proteins are key regulators of many biotic, abiotic and physiological responses in plants (Phukan et al., 2016). Similarly, the ORFs within the gene sequence of Ha6g003560 were confirmed with NCBI ORFfinder. The confirmed ORF sequence was used for BLASTp searches to obtain the most similar protein sequences in other phyla (Figure S6). As shown in the phylogenetic tree, Ha6g003560 closely resembles *Arabidopsis thaliana* gene *WRKY44/TTG2*. It has been shown that the *WRKY44/TTG2* gene regulates trichome development in Arabidopsis (Ishida et al., 2007). In addition, we also performed alignments with the genomic sequence of gene Ha6_7633946 from HA 300, RHA 273 and reference genome XRQ. Since the sequence quality was poor in this region in RHA 464 we used the sequence from RHA 273 instead because these two inbred lines are phylogenetically close, they share the same phenotype for CGT, and they share the same haplotype of chromosome 6 (Table 3, Figure S4). As a result, we found a 51 bp deletion in the intron of gene Ha6_7633946 in RHA 273, and this deletion results in an alternative splicing (Figures 5B, 6). These results make the putative HSF gene Ha5g003120 and WRKY gene Ha6g003560 good candidates as the regulators of CGT density in sunflower florets.

## Discussion

Trichomes, especially glandular trichomes, play an important role in plant defense. CGTs are most common in Asteraceae species (Bombo et al., 2016). CGTs are well-known for their ability to produce various secondary metabolites such as STLs. In sunflower, however, the genetics of CGT density is not well understood. In this study, we reported for the first time that CGT density in sunflower florets is a quantitative trait, and two major QTL were identified in a biparental mapping population. The F_4_ mapping population derived from high CGT density parent HA 300 and low CGT density parent RHA 464, showed segregation in CGT number per floret (Figure 1). Of 179 F_4_ plants, only 25 plants (13.9%) showed high CGT density (greater than 150 CGT per floret) and 45 plants (25.2%) showed low CGT density (less than 50 CGT per floret). The variation of CGT density from CGT counting errors was marginal since the CGT numbers from first floret and second floret were highly correlated (Figure S1). As shown in Figure 1, the frequency of CGT density in the F_4_ mapping population showed a moderately skewed distribution (Skewness = 0.98) toward the low CGT density parent. For this reason, we attempted non-parametric interval mapping, which is an alternative method for analyzing non-normal phenotypic data, but it exhibited less power to detect QTL in our population (Broman, 2003; Fernandes et al., 2007; data not shown). In general, the simple interval mapping method can give reasonable results if the phenotypic data are not highly skewed (Broman and Sen, 2009). We also determined statistical significance on the basis of a genome-wide permutation test, which utilizes the same empirical distribution (Table S3). For these reasons, we used the original CGT numbers to perform QTL analysis without any data transformation.

The linkage map in the QTL analysis was constructed with SNP markers generated through GBS. GBS is a high-throughput and highly cost-effective genotyping and SNP discovery approach, and has been applied to many plant species (Elshire et al., 2011; Kumar et al., 2012; Melo et al., 2016; Torkamaneh et al., 2016). To date, two GBS-SNP marker maps have been reported in sunflower (Celik et al., 2016; Talukder et al., 2016). In our study, we successfully mapped 2121 SNP markers, which is more than twice the number of unique SNP markers mapped in previous studies (Table 1). There are two possible reasons to explain why we were able to discover more unique SNP markers by GBS. First, we used a customized bioinformatic pipeline to process the GBS data. As described in our methods, we followed the GATK Best Practices to recalibrate the alignment, and used FreeBayes, a haplotype-based variant caller, to detect SNP markers (Garrison and Marth, 2012). The pipeline we used in our study could be valuable for discovering SNP markers, in addition to the TASSEL-GBS pipeline which was used in previous studies (Glaubitz et al., 2014; Celik et al., 2016; Talukder et al., 2016). Second, for our biparental mapping population, we carried out whole genome sequencing for the parent lines (HA 300 and RHA 464). These were included in the SNP marker calling and imputation steps together with their offspring, and this greatly helped to discover more reliable SNP markers. It is also a good practice to extract DNA from fresh young leaves or seedlings to obtain high quality DNA for GBS. With these modifications, we think GBS is still a good option for genotyping and SNP discovery in sunflower, especially considering the advantage of high-throughput and cost-effectiveness. The length of the genetic linkage map in this study was 1,519.52 cM, which is comparable with a sunflower consensus linkage map of 1,443.84 cM developed from three F2 mapping populations (Talukder et al., 2014), and also a recent SNP-based linkage map of 1,401.36 cM (Talukder et al., 2016). The marker density in this study (0.72 cM/SNP) is higher than the other two SNP marker maps (1.33 cM/SNP; Talukder et al., 2016; 3.03 cM/SNP; Celik et al., 2016). Despite good whole genome coverage, several regions with gaps greater than 10 cM were observed on chromosome 4, 6, and 14 (Table 1). Notably, the largest gap is 27.32 cM on chromosome 6, which is likely due to chromosome structure differences between the parents. Consequently, we were unable to detect SNP markers from these regions.

Two major QTL controlling CGT density in sunflower were identified in a biparental mapping population (Figure 3). One QTL was mapped to chromosome 5 (Ha5@14.6), which explained 11.61% of the CGT number variation. The second QTL was mapped to chromosome 6 (Ha6@60.5), which explained 14.06% of the CGT number variation (Table 2). No epistasis interaction was detected, and this suggests that these two QTL mediate CGT density in an additive manner. Similarly, two QTL associated with type VI glandular trichome density were identified in cultivated tomato, and seven QTL associated with trichome density were detected in soybean (Maliepaard et al., 1995; Du et al., 2009). These findings suggest that the trichome density in plants is regulated by multiple genes. A large portion of the CGT number variation is still unaccounted for in the current study, and this could be due to some error in genotyping, or a confluence of several genes of minor effect.

To validate the QTL results, we selected 39 sunflower inbred lines with diverse genetic backgrounds as the validation population (Figure S4). We used multiple linear regression analysis to validate the QTL-trait association and also estimate the QTL effects. A total of 385 SNP markers were selected from the 1.5-LOD support interval on chromosome 5 and 33 SNP markers were selected from the QTL support interval on chromosome 6. Based on annotated gene functions, one SNP marker pair, Ha5_10149906 and Ha6_7633946, was identified as the best two-QTL model and corresponded to genes with a plausible effect on the phenotype. This two-QTL model explained 47.2% of phenotypic variation in the validation population. Moreover, the significant associations between single SNP markers (Ha5_10149906 and Ha6_7633946) and CGT number were also supported by single factor ANOVA (Figures 4C,D). Thus, the QTL effects and the association between CGT density and QTL support intervals were confirmed in validation.

The SNP marker Ha5_10149906 is located within the second exon of the gene Ha5g003120. A 326 bp deletion was observed in the promoter region of the gene Ha5g003120 in RHA 464, and this deletion might lead to substantial change in gene expression pattern (Figure 5A). The gene Ha5g003120 is annotated as a member of heat shock transcription factor (HSF) family. Although no experimental results have shown that HSF proteins are involved in trichome development, some studies indicate these proteins are required for cell division and root growth (Westerheide et al., 2012). The SNP marker Ha6_7633946 is located in the 3′-UTR of the gene Ha6g003560 and is associated with an alternative splice site in RHA 273, due to a 51 bp deletion in the second intron. This alternative splicing alters the *WRKY* domain, which determines DNA-binding specificity (Llorca et al., 2014). The phylogenetic tree showed that Ha6g003560 is grouped together with *Arabidopsis thaliana* gene *WRKY44/TTG2. WRKY44/TTG2* is a key regulatory gene for trichome development, and a mutation in *WRKY44/TTG2* causes significantly reduced trichome number and unbranched trichomes in Arabidopsis (Johnson, 2002; Ishida et al., 2007; Pesch et al., 2014). Taken together, the *HSF* gene Ha5g003120 and *WRKY* gene Ha6g003560 are strong candidates for regulating CGT density in sunflower florets. It is possible, however, that other adjacent genes are contributing to the phenotype. Further studies are required to characterize gene functions in these regions.

In summary, we successfully detected SNP markers by GBS and constructed a genetic linkage map with these SNP markers. We also identified two major QTL controlling CGT density in sunflower florets by using the F_4_ population derived from the cross HA 300 × RHA 464. In addition, we found two plausible candidate genes in the QTL support intervals. Future work will focus on optimizing STL chemical composition in CGT for enhancing host resistance to sunflower insect pests.

## Author note

Mention of trade names or commercial products in this report is solely for the purpose of providing specific information and does not imply recommendation or endorsement by the US Department of Agriculture. The USDA is an equal opportunity provider and employer.

## Author contributions

Q-MG analyzed the data and wrote the paper. NK, BH, and JP designed and led the genomics analyses, population development and genetics analyses, and trait evaluations, respectively. SR conducted phylogenetic analyses of the candidate genes. CP and ST conducted GBS and whole genome shotgun sequencing and bioinformatic analyses. All authors have read and agree with the contents of the manuscript.

### Conflict of interest statement

The authors declare that the research was conducted in the absence of any commercial or financial relationships that could be construed as a potential conflict of interest.
