# Supplementary material for: Genetic Architecture of Capitate Glandular Trichome Density in Florets of Domesticated Sunflower (Helianthus annuus L.)
Source: Front Plant Sci. 2018 Jan 9;8:2227. doi: 10.3389/fpls.2017.02227 (PMC5767279; doi:10.3389/fpls.2017.02227)
Supplement: Supplementary file 1 [file Table1.PDF]

Table S1. Cultivars used for single copy site filtering.

Cultivar	USDA-GRIN Accession	Cultivar type
Hopi	PI369359	Landrace
NMS373	PI597362	Modern cultivar
RHA358	PI531071	Modern cultivar
SF33	N/A	Modern cultivar
RHA415	PI607506	Modern cultivar
