# Supplementary material for: Genetic Architecture of Capitate Glandular Trichome Density in Florets of Domesticated Sunflower (Helianthus annuus L.)
Source: Front Plant Sci. 2018 Jan 9;8:2227. doi: 10.3389/fpls.2017.02227 (PMC5767279; doi:10.3389/fpls.2017.02227)
Supplement: Supplementary file 2 [file Table2.pdf]

Table S2. Maximum LOD values for each chromosome from the one QTL scan in the F_4 mapping population of HA 300 x RHA 464.

Chromosome	Position (cM)	LOD
1	15.93	0.52
2	28.38	0.67
3	0.62	1.93
4	56.59	1.16
5	14.64	5.02
6	60.72	5.98
7	4.77	0.63
8	22.02	2.34
9	61.24	1.67
10	76.68	1.20
11	40.98	1.15
12	52.05	1.01
13	5.04	2.64
14	18.10	1.08
15	57.50	0.87
16	68.61	1.47
17	72.63	2.03
