# Supplementary material for: Genetic Architecture of Capitate Glandular Trichome Density in Florets of Domesticated Sunflower (Helianthus annuus L.)
Source: Front Plant Sci. 2018 Jan 9;8:2227. doi: 10.3389/fpls.2017.02227 (PMC5767279; doi:10.3389/fpls.2017.02227)
Supplement: Supplementary file 3 [file Table3.pdf]

Table S3. Summary of two-QTL scan LOD scores and genome-wide permutation threshold for full two-QTL model (T_f), full two-QTL model over a single QTL model (T_{fv1}), additive model (T_a), interaction model (T_i), additive model over a single QTL model (T_{av1}).

	T_f	T_{fv1}	T_a	T_i	T_{av1}
LOD threshold (α = 0.05, 1000 permutation)	10.46	7.57	7.65	6.32	4.6
Ha5@14.6 : Ha6@60.5 two QTL scan	11.9	5.86	10.8	1.1	4.76
