# Supplementary material for: Genetic Architecture of Capitate Glandular Trichome Density in Florets of Domesticated Sunflower (Helianthus annuus L.)
Source: Front Plant Sci. 2018 Jan 9;8:2227. doi: 10.3389/fpls.2017.02227 (PMC5767279; doi:10.3389/fpls.2017.02227)
Supplement: Supplementary file 4 [file Table4.pdf]

Table S4. Sequences of two significant SNP markers identified in the HA 300 × RHA 464 mapping population.

SNP marker	Allele	Map position (cM)	SNP sequence
Ha5_11356218	C/T[a]	14.6	AGTGGCTTTCACATATGGTCATAAAGTTCTAATACTTCTTGGGAGATCTTTTCTGTTACTAATC[C/T]TTTCATCTGCAACTGACTATTTGGACAGTGAATTCTATTCGGTTACTCAGCTGATAATAATGTTTATACCATAGTAGTTGAAC
Ha6_8364901	T/C[a]	60.5	TTTAAATGTCCACTTGCTTTGAACAACACTCCATGGCCTGCGGTACC[T/C]TTTAGATACCTTAGAATTCTCTGAGCAGCTTCCATGTGGGCAACTTGCGGTTGGTGCATAAACTGACTTACCACTCC

[a] The first allele is from HA 300 and the second allele is from RHA 464.
