# Supplementary material for: Genetic Architecture of Capitate Glandular Trichome Density in Florets of Domesticated Sunflower (Helianthus annuus L.)
Source: Front Plant Sci. 2018 Jan 9;8:2227. doi: 10.3389/fpls.2017.02227 (PMC5767279; doi:10.3389/fpls.2017.02227)
Supplement: Supplementary file 5 [file Table5.PDF]

Table S5. A list of predicted genes in two QTL support intervals.

Gene ID	Putative function	Physical position	SNP marker	Expressed in flower tissues[a]
Ha5g002680	DNA-binding pseudobarrel domain	Ha5:8067303-8069892	/	Yes
Ha5g002690	Zinc finger, RING/FYVE/PHD-type	Ha5:8073459-8074580	Ha5_8076617	Yes
Ha5g002700	Ubiquitin conjugation factor E4	Ha5:8081716-8082381	/	Yes
Ha5g002710	Unkown	Ha5:8232302-8233837	/	No
Ha5g002720[b]	Reticulon	Ha5:8386875-8387579	Ha5_8384389	Yes
Ha5g002730[b]	Concanavalin A-like lectin/glucanase	Ha5:8413735-8415588	Ha5_8412226	Yes
Ha5g002740	Glycoside hydrolase	Ha5:8429516-8432635	/	Yes
Ha5g002750	S-locus glycoprotein	Ha5:8455670-8456955	Ha5_8454100	Yes
Ha5g002760	Serine/threonine- / dual specificity protein kinase	Ha5:8515933-8517502	/	Yes
Ha5g002770	Cupredoxin	Ha5:8537308-8537836	/	No
Ha5g002780	Unkown	Ha5:8565985-8566782	/	No
Ha5g002790[b]	S-locus glycoprotein	Ha5:8809904-8811512	Ha5_8809492	Yes
Ha5g002800	Unkown	Ha5:8780599-8780977	/	No
Ha5g002810	Unkown	Ha5:8781815-8784638	/	Yes
Ha5g002820	S-locus glycoprotein	Ha5:8809904-8811512	/	No
Ha5g002830	Concanavalin A-like lectin/glucanase	Ha5:8811832-8813590	Ha5_8811812	No

Ha5g002840	S-locus glycoprotein	Ha5:8819362 -8820690	Ha5_882116 5	No
Ha5g002850	Serine/threonine-/dual specificity protein kinase	Ha5:8821445 -8823234	Ha5_882330 9	No
Ha5g002860	Unkown	Ha5:8869347 -8870270	/	Yes
Ha5g002870	Transposase (putative), gypsy type	Ha5:8991985 -8994519	/	No
Ha5g002880	Zinc finger, RING/FYVE/PHD-type	Ha5:9068497 -9068811	/	Yes
Ha5g002890	Copper chaperone SCO1/SenC	Ha5:9151371 -9152474	/	Yes
Ha5g002900	Unkown	Ha5:9354746 -9356001	/	No
Ha5g002910	Unkown	Ha5:9488476 -9489491	/	No
Ha5g002920	Macro domain	Ha5:9492321 -9500388	/	Yes
Ha5g002930	Amino acid/polyamine transporter I	Ha5:9546184 -9546708	/	No
Ha05g00294 0[b]	Unknown	Ha5:9553185 -9554523	Ha5_955246 8	Yes
Ha5g002950	Unkown	Ha5:9558182 -9560601	/	Yes
Ha5g002960[b]	PapD-like	Ha5:9640099 -9641710	Ha5_964015 6	Yes
Ha5g002970	Unkown	Ha5:9660062 -9660429	/	Yes
Ha5g002980	Unkown	Ha5:9724995 -9726269	/	
Ha5g002990[b]	PapD-like	Ha5:9735653 -9738037	Ha5_973357 4	Yes

Ha5g003000[b]	Peptidoglycan-binding lysin domain	Ha5:9744885-9745348	Ha5_9744793	No
Ha5g003010	Unkown	Ha5:9771113-9771289	/	Yes
Ha5g003020	Isopenicillin N synthase-like	Ha5:9793888-9800555	Ha5_9796474	No
Ha5g003030	Oxoglutarate/iron-dependent dioxygenase	Ha5:9834392-9839383	Ha5_9834977	No
Ha5g003040[b]	Protein of unknown function DUF616	Ha5:9853932-9859320	Ha5_9853932	Yes
Ha5g003050	Unkown	Ha5:9859877-9859997	/	No
Ha5g003060	Protein of unknown function DUF616	Ha5:9862110-9862289	/	Yes
Ha5g003070[b]	Glycoside hydrolase	Ha5:9897724-9898634	Ha5_9897749	No
Ha5g003080	General substrate transporter	Ha5:9933389-9936007	/	Yes
Ha5g003090	SAM dependent carboxyl methyltransferase	Ha5:10004696-10005102	/	No
Ha5g003100	Glycosyl transferase	Ha5:10137741-10139355	Ha5_10136915	Yes
Ha5g003110	Proton-dependent oligopeptide transporter family	Ha5:10147204-10147630	/	Yes
Ha5g003120[b]	Winged helix-turn-helix DNA-binding domain	Ha5:10149384-10150305	Ha5_10149906	No
Ha5g003130	Heat shock protein 70 family	Ha5:10235097-10238054	/	Yes
Ha5g003140	Unkown	Ha5:10264056-10264216	/	No
Ha5g003150	Protein kinase C-like	Ha5:10268401-10270522	/	No

Ha5g003160	Protein of unknown function DUF617	Ha5:10338149-10338916	/	Yes
Ha5g003170	Heat shock protein 70 family	Ha5:10339528-10341460	/	Yes
Ha5g003180	Heat shock protein 70 family	Ha5:10343996-10344820	/	Yes
Ha5g003190	Six-bladed beta-propeller, TolB-like	Ha5:10364149-10366326	/	Yes
Ha5g003200	Unkown	Ha5:10407518-10408185	/	No
Ha5g003210	P-loop containing nucleoside triphosphate hydrolase	Ha5:10408264-10408584	/	No
Ha5g003220	Uncharacterised protein family Ycf2	Ha5:10420467-10420955	/	No
Ha5g003230	Ubiquitin supergroup	Ha5:10427047-10427757	/	No
Ha5g003240	Unkown	Ha5:10433036-10435287	/	No
Ha5g003250	Cysteine peptidase	Ha5:10453664-10454903	/	No
Ha5g003260	HAD hydrolase, subfamily IA	Ha5:10463684-10464365	/	Yes
Ha5g003270	Riboflavin kinase	Ha5:10498876-10500070	/	No
Ha5g003280	Cysteine peptidase	Ha5:10505490-10506707	/	No
Ha5g003290	Unkown	Ha5:10547307-10548263	/	No

Ha5g003300	Ubiquitin supergroup	Ha5:10564187-10565098	/	No
Ha5g003310	Unkown	Ha5:10682699-10683716	/	Yes
Ha5g003320	F-box domain	Ha5:10685799-10686943	/	Yes
Ha5g003330	Gnk2-homologous domain	Ha5:10836850-10837614	/	No
Ha5g003340	Unkown	Ha5:10840966-10842948	/	No
Ha5g003350	Unkown	Ha5:10845967-10846185	/	No
Ha5g003360	Unkown	Ha5:10890285-10896233	/	No
Ha5g003370	Glutamyl/glutaminyl-tRNA synthetase	Ha5:10896235-10905638	/	Yes
Ha5g003380	C1-like protein	Ha5:10969643-10970491	/	Yes
Ha5g003390	C1-like protein	Ha5:10985002-10985850	/	Yes
Ha5g003400	C1-like protein	Ha5:10987051-10988403	/	Yes
Ha5g003410	C1-like protein	Ha5:11049908-11051044	/	No
Ha5g003420	Unkown	Ha5:11100825-11105458	/	Yes
Ha5g003430	Unkown	Ha5:11127049-11129262	/	Yes

Ha5g003440	Unkown	Ha5:11129770-11130434	/	Yes
Ha5g003450	WD40/YVTN repeat-like-containing domain	Ha5:11130436-11131275	/	Yes
Ha5g003460	Unkown	Ha5:11131277-11131553	/	No
Ha5g003470	Nucleosome assembly protein	Ha5:11131555-11133140	/	Yes
Ha5g003480	Photosystem II PsbP	Ha5:11159776-11160666	/	Yes
Ha5g003490	Photosystem II PsbP	Ha5:11160923-11161378	/	Yes
Ha5g003500	Unkown	Ha5:11167736-11169466	/	No
Ha5g003510	EF-hand domain pair	Ha5:11171329-11176579	/	Yes
Ha5g003520	Unkown	Ha5:11176653-11181097	/	No
Ha5g003530[b]	Unkown	Ha5:11194158-11194852	Ha5_11194388	No
Ha5g003540	WD40/YVTN repeat-like-containing domain	Ha5:11261369-11269269	/	No
Ha5g003550	Unkown	Ha5:11289144-11291632	/	Yes
Ha5g003560	Bifunctional 3-hexulose-6-phosphate synthase	Ha5:11309561-11311698	/	No

Ha5g003570	Glutaredoxin-like	Ha5:11330866-11331276	/	No
Ha5g003580	Cyclophilin-like peptidyl-prolyl cis-trans isomerase	Ha5:11338195-11340464	/	Yes
Ha5g003590	ACT domain	Ha5:11342268-11345028	/	No
Ha5g003600	Peptidase S8	Ha5:11351419-11352171	/	No
Ha5g003610	Glycoside hydrolase	Ha5:11360886-11364205	Ha5_11360355	No
Ha5g003620	Pectin lyase fold/virulence factor	Ha5:11391213-11392393	/	No
Ha5g003630	Unkown	Ha5:11426809-11427487	/	NO
Ha5g003640[b]	Proton-dependent oligopeptide transporter family	Ha5:11470487-11473096	Ha5_11469460	Yes
Ha5g003650	Serine/threonine- / dual specificity protein kinase,	Ha5:11477886-11480821	/	No
Ha5g003660	Unkown	Ha5:11483647-11483979	/	Yes
Ha5g003670	Myosin head, motor domain	Ha5:11506878-11510151	/	Yes
Ha5g003680	Myosin head, motor domain	Ha5:11577457-11578267	/	Yes

Ha5g003690	Zinc finger, PHD-type	Ha5:11583524-11584387	/	No
Ha5g003700	Lipoxygenase	Ha5:11651323-11653045	/	No
Ha5g003710	EF-hand domain pair	Ha5:11733608-11734105	/	Yes
Ha5g003720[b]	DNA-dependent epimerase/dehydratase	Ha5:11742498-11743332	Ha5_11740223	No
Ha5g003730	NAD(P)-binding domain	Ha5:11770054-11770329	/	Yes
Ha5g003740	Unkown	Ha5:11801837-11802709	/	No
Ha5g003750	Unkown	Ha5:11817413-11818593	/	Yes
Ha5g003760	Glucose/ribitol dehydrogenase	Ha5:11854068-11855424	/	Yes
Ha5g003770	Photosynthetic reaction centre, L/M	Ha5:11858241-11858420	/	No
Ha5g003780	Remorin	Ha5:11892739-11893802	Ha5_11893514	Yes
Ha5g003790	Glucose/ribitol dehydrogenase	Ha5:11972374-11973953	/	Yes
Ha5g003800	Glutamate/phenylalanine/leucine/valine dehydrogenase	Ha5:11995100-11998411	/	Yes
Ha5g003810	SRA-YDG	Ha5:11999322-12000617	/	No
Ha5g003820	Peptide methionine sulphoxide reductase MsrA	Ha5:12007347-12009948	/	Yes
Ha5g003830	Short-chain dehydrogenase/reductase SDR	Ha5:12139374-12139634	/	Yes
Ha5g003840[b]	Zona occludens protein ZO-3	Ha5:12161253-12162362	Ha5_12161345	Yes

Ha5g003850[b]	Guanylate kinase/L-type calcium channel beta subunit	Ha5:12238965-12239917	Ha5_12237038	Yes
Ha5g003860	Unkown	Ha5:12292295-12292428	/	No
Ha5g003870	Unkown	Ha5:12292512-12292649	/	No
Ha5g003880	Unkown	Ha5:12332845-12332982	/	No
Ha5g003890[b]	D-aminoacyl-tRNA deacylase	Ha5:12395168-12398448	Ha5_12395245	Yes
Ha5g003900	Unkown	Ha5:12402769-12402929	/	No
Ha5g003910	Unkown	Ha5:12403224-12403373	/	No
Ha5g003920	DNA recombination and repair protein Rad51	Ha5:12405006-12406273	/	Yes
Ha5g003930	G-box binding, MFMR	Ha5:12492009-12492453	/	Yes
Ha5g003940	Diacylglycerol kinase	Ha5:12511731-12512502	/	Yes
Ha5g003950	Unkown	Ha5:12524101-12524248	/	No
Ha5g003960	C-terminal-processing peptidase S41A	Ha5:12593496-12596841	/	Yes
Ha5g003970	Zinc finger, RING/FYVE/PHD-type	Ha5:12598130-12602693	Ha5_12603257	Yes
Ha5g003980[b]	DnaJ domain	Ha5:12613948-12615607	Ha5_12613123	Yes
Ha6g003490	RNA helicase	Ha6:7496840-7499966	/	Yes
Ha6g003500	Unkown	Ha6:7514747-7516769	/	Yes

Ha6g003510	Unkown	Ha6:7521009-7522762	/	Yes
Ha6g003520	Unkown	MULE transposase domain	/	Yes
Ha6g003530[c]	DNA-binding WRKY	Ha6:7535068-7535610	Ha6_7534816	Yes
Ha6g003540	Unkown	Ha6:7590506-7592320	/	Yes
Ha6g003550	Unkown	Ha6:7606538-7610391	/	Yes
Ha6g003560[c]	DNA-binding WRKY	Ha6:7634391-7636099	Ha6_7633946	No
Ha6g003570	Unkown	Ha6:7643938-7644573	/	No
Ha6g003580[c]	Glycoside hydrolase	Ha6:7647852-7648932	Ha6_7650962	Yes
Ha6g003590	Helicase	Ha6:7654881-7657085	Ha6_7655637	Yes
Ha6g003600	Glucose/ribitol dehydrogenase	Ha6:7663482-7672056	/	Yes
Ha6g003610	Translation initiation factor 3 complex subunit L;	Ha6:7680558-7682224	/	Yes
Ha6g003620	DNA-binding WRKY	Ha6:7685564-7685860	/	No
Ha6g003630	Zinc finger, MIZ-type	Ha6:7734757-7742703	/	Yes
Ha6g003640	Nucleotide-binding, alpha-beta plait	Ha6:7750286-7753722	/	Yes
Ha6g003650	Unkown	Ha6:7756298-7760513	/	Yes
Ha6g003660	Unkown	Ha6:7782845-7783132	/	No
Ha6g003670	Reticulon	Ha6:7798591-7801199	/	Yes

Ha6g003680	P-loop containing nucleoside triphosphate hydrolase	Ha6:7802379-7803700	/	No
Ha6g003690	P-loop containing nucleoside triphosphate hydrolase	Ha6:7804323-7805092	/	No
Ha6g003700	Unkown	Ha6:7807615-7808302	/	No
Ha6g003710	Unkown	Ha6:7828730-7831138	/	Yes
Ha6g003720	Glycoside hydrolase	Ha6:7832356-7833993	/	No
Ha6g003730	Glycoside hydrolase	Ha6:7838240-7840201	/	No
Ha6g003740	Unkown	Ha6:7860006-7860606	/	Yes
Ha6g003750	Unkown	Ha6:7879888-7882500	/	Yes
Ha6g003760	Unkown	Ha6:7941965-7943332	/	Yes
Ha6g003770	Cytochrome b245	Ha6:7977417-7986898	/	Yes
Ha6g003780	Unkown	Ha6:8040789-8041438	/	Yes
Ha6g003790	Cation/H+ exchanger	Ha6:8047040-8050146	/	Yes
Ha6g003800	Unkown	Ha6:8061471-8062619	/	Yes
Ha6g003810	Unkown	Ha6:8068760-8068837	/	No
Ha6g003820	Unkown	Ha6:8074436-8074741	/	No
Ha6g003830	Unkown	Ha6:8078047-8078538	/	Yes
Ha6g003840[c]	Enolase	Ha6:8083694-8088034	Ha6_8089162	Yes
Ha6g003850	Unkown	Ha6:8091926-8092405	/	No

Ha6g003860	Unkown	Ha6:8135400 -8137227	/	No
Ha6g003870	Acetyl-CoA carboxylase carboxyl transferase	Ha6:8139099 -8140957	/	Yes
Ha6g003880	Ribosomal protein L5	Ha6:8141216 -8141779	/	Yes
Ha6g003890	Photosystem I PsaA/PsaB	Ha6:8142142 -8143603	/	Yes
Ha6g003900	Unkown	Ha6:8223255 -8223570	/	Yes
Ha6g003910[c]	Zinc finger, C2H2	Ha6:8268763 -8269614	Ha6_8270588	No
Ha6g003920	Development/cell death domain	Ha6:8377094 -8377558	/	Yes
Ha6g003930	Development/cell death domain	Ha6:8384024 -8384286	/	No
Ha6g003940	Dynamin superfamily	Ha6:8387984 -8392081	/	No
Ha6g003950	Unkown	Ha6:8413015 -8413090	/	No
Ha6g003960	Unkown	Ha6:8413653 -8418543	/	Yes
Ha6g003970	Unkown	Ha6:8419398 -8421241	/	No
Ha6g003980	Unkown	Ha6:8421342 -8421453	/	No
Ha6g003990	Unkown	Ha6:8421545 -8421686	/	No
Ha6g004000	Unkown	Ha6:8421871 -8421946	/	No
Ha6g004010	Alpha/beta hydrolase	Ha6:8432832 -8437182	/	Yes
Ha6g004020	Unkown	Ha6:8463900 -8466198	/	Yes

Ha6g004030	Unkown	Ha6:8466757-8468402	/	Yes
Ha6g004040	Translation elongation/initiation factor	Ha6:8504151-8506066	/	Yes
Ha6g004050	Sucrose synthase	Ha6:8557832-8563651	/	Yes
Ha6g004060	Citrate synthase-like	Ha6:8568546-8571401	/	Yes

[a] The expression data obtained from RNAseq is available at https://www.heliagene.org.
[b] The genes on chromosome 5 showed significant association with CGT density in the validation population.
[c] The genes on chromosome 6 showed significant association with CGT density in the validation population.
/ Represents missing data.
