# Supplementary material for: Genetic Architecture of Capitate Glandular Trichome Density in Florets of Domesticated Sunflower (Helianthus annuus L.)
Source: Front Plant Sci. 2018 Jan 9;8:2227. doi: 10.3389/fpls.2017.02227 (PMC5767279; doi:10.3389/fpls.2017.02227)
Supplement: Supplementary file 6 [file Presentation1.pdf]

Supplementary Material

Genetic architecture of capitate glandular trichome density in florets of domesticated sunflower (Helianthus annuus L.)

Q-M. Gao, N.C. Kane, B.S. Hulke*, S. Reinert, C. S. Pogoda, S. Tittes, and J.R. Prasifka

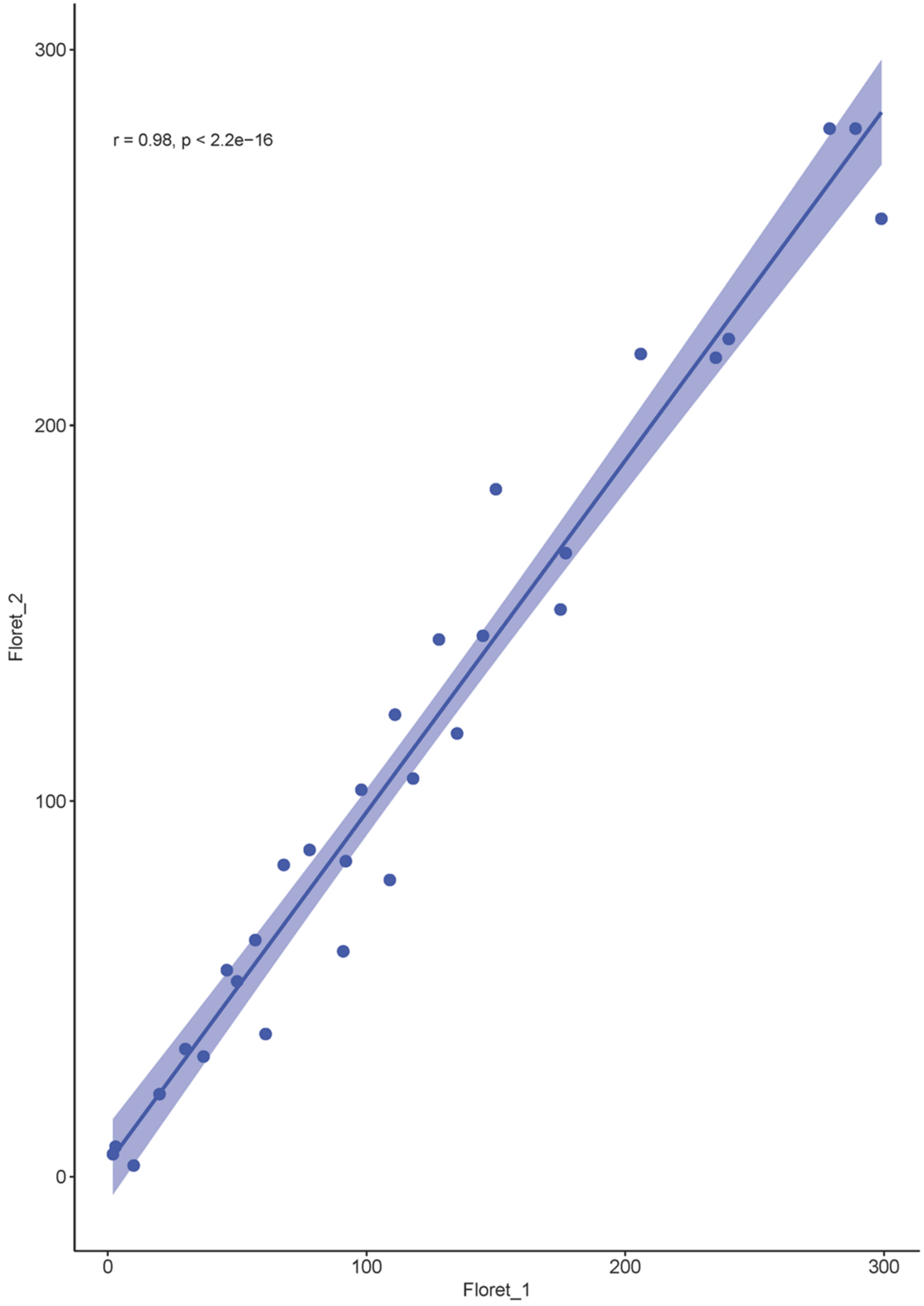

Figure S1. Within plant correlation of CGT counts from 30 plants in the F4 mapping population. The CGT numbers from first floret and second floret were highly correlated (r = 0.98, $p < 2.2 \times 10^{-16}$).

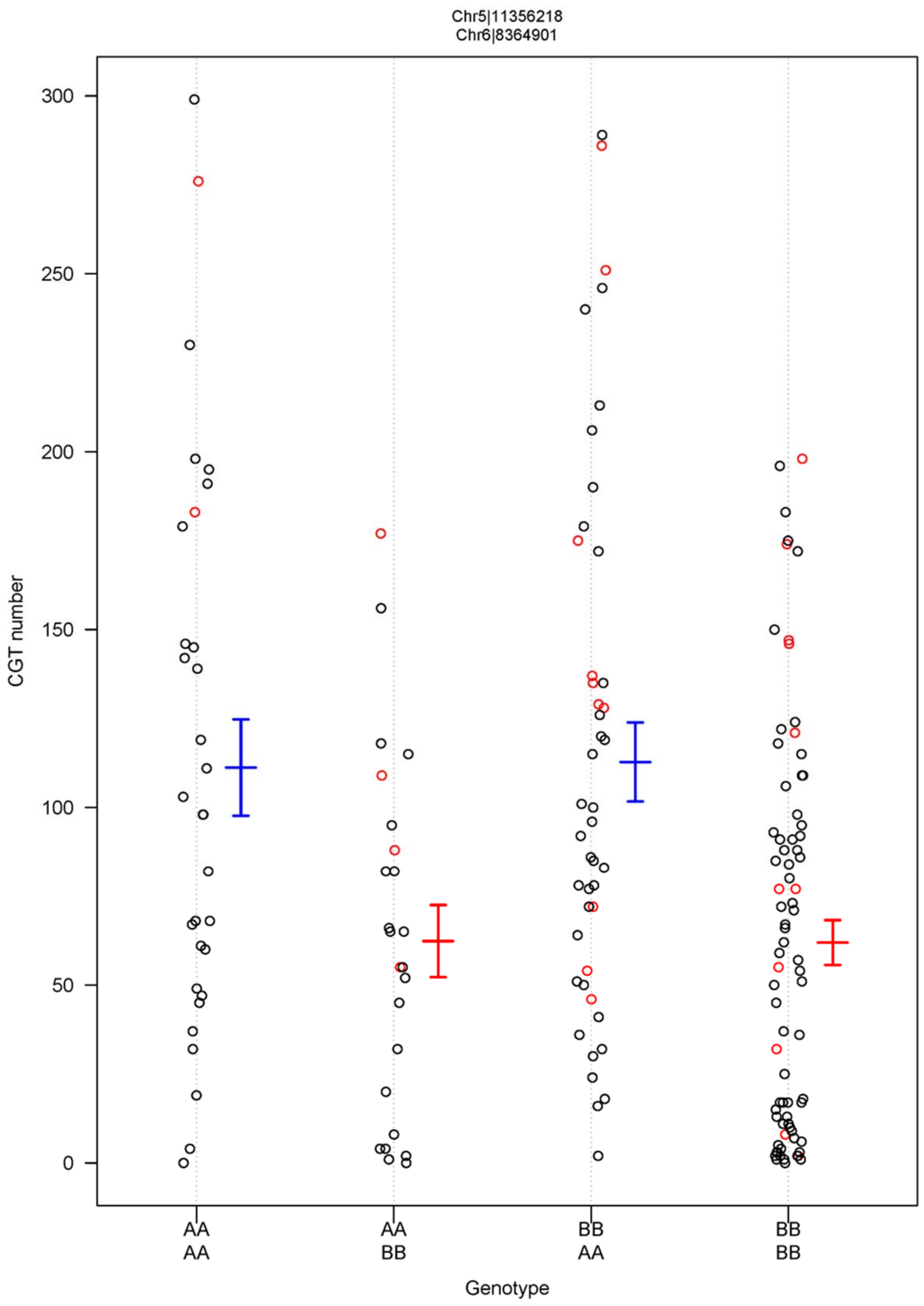

Figure S2. Plot of CGT number against marker genotypes at SNP markers Ha5_11356218 and Ha6_8364901 in the F4 mapping population. Error bars are ± 1 SE.

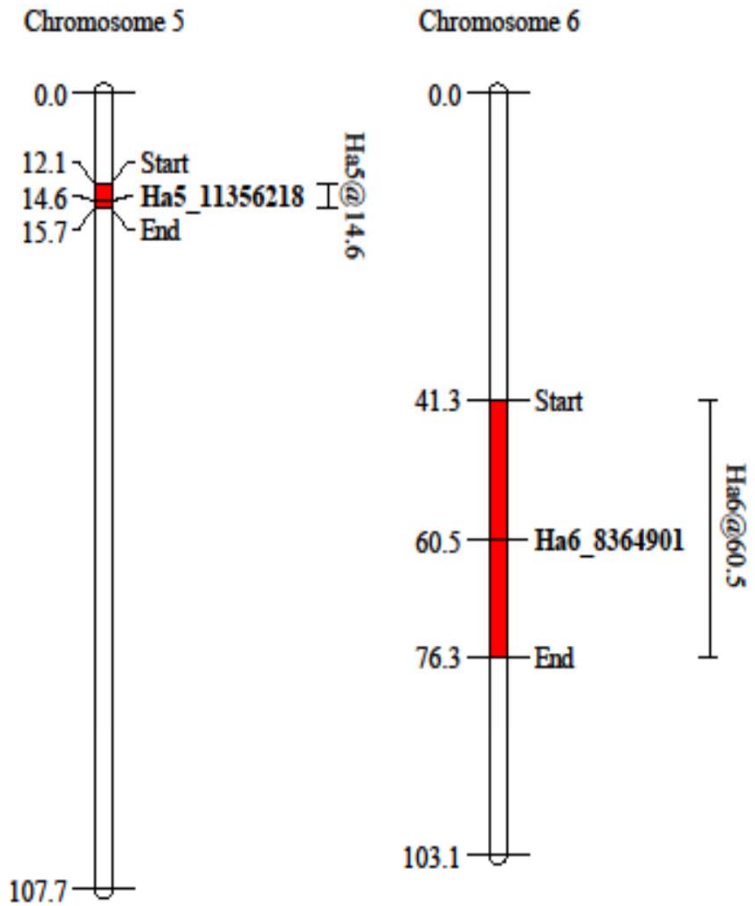

Figure S3. Map positions of two significant QTL intervals identified in the F4 mapping population derived from HA300 × RHA464.

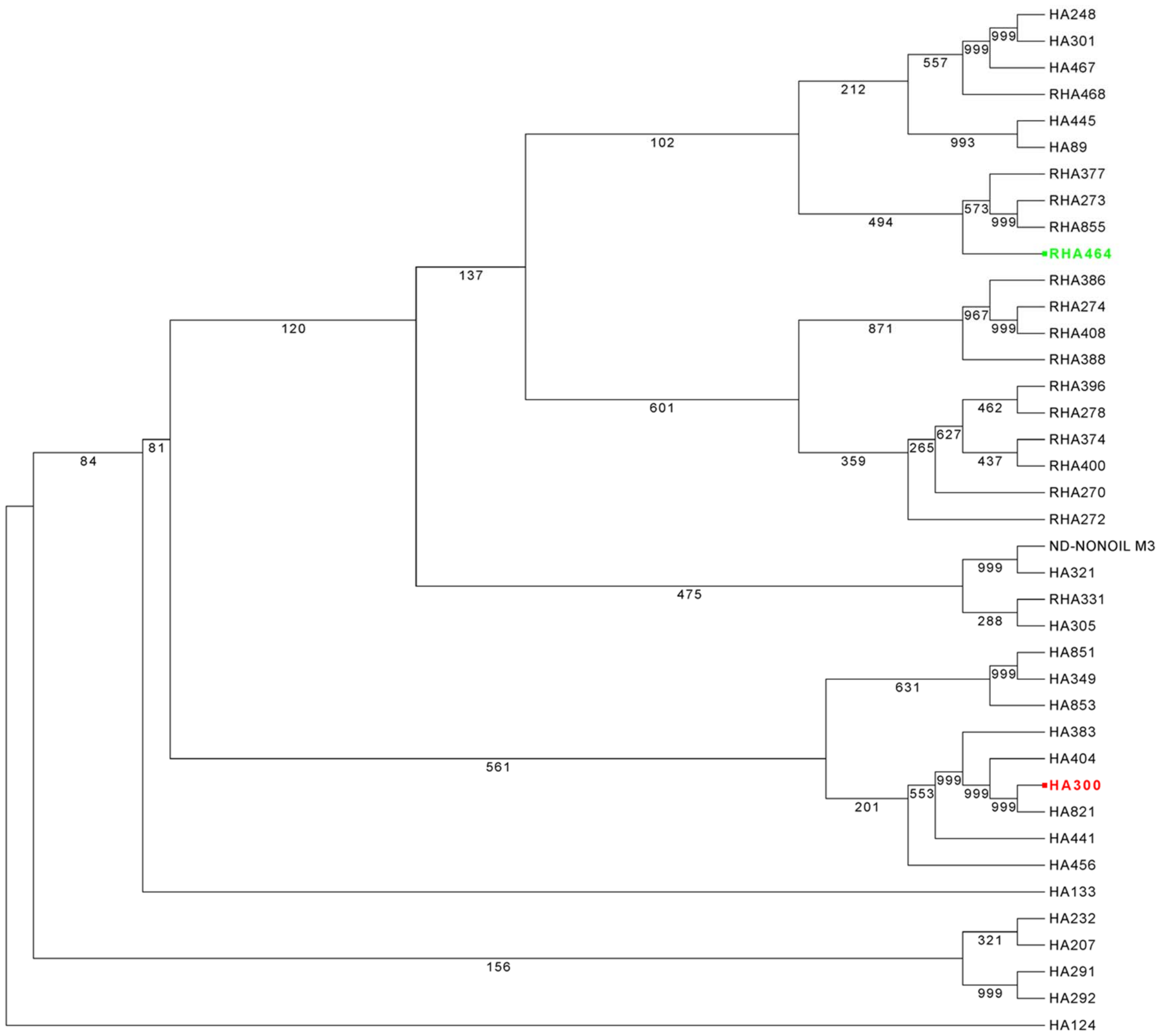

Figure S4. Phylogenetic tree of the validation population generated from SNP marker data. Nodes are labeled with their bootstrap support values. The two parental lines are indicated in colors.

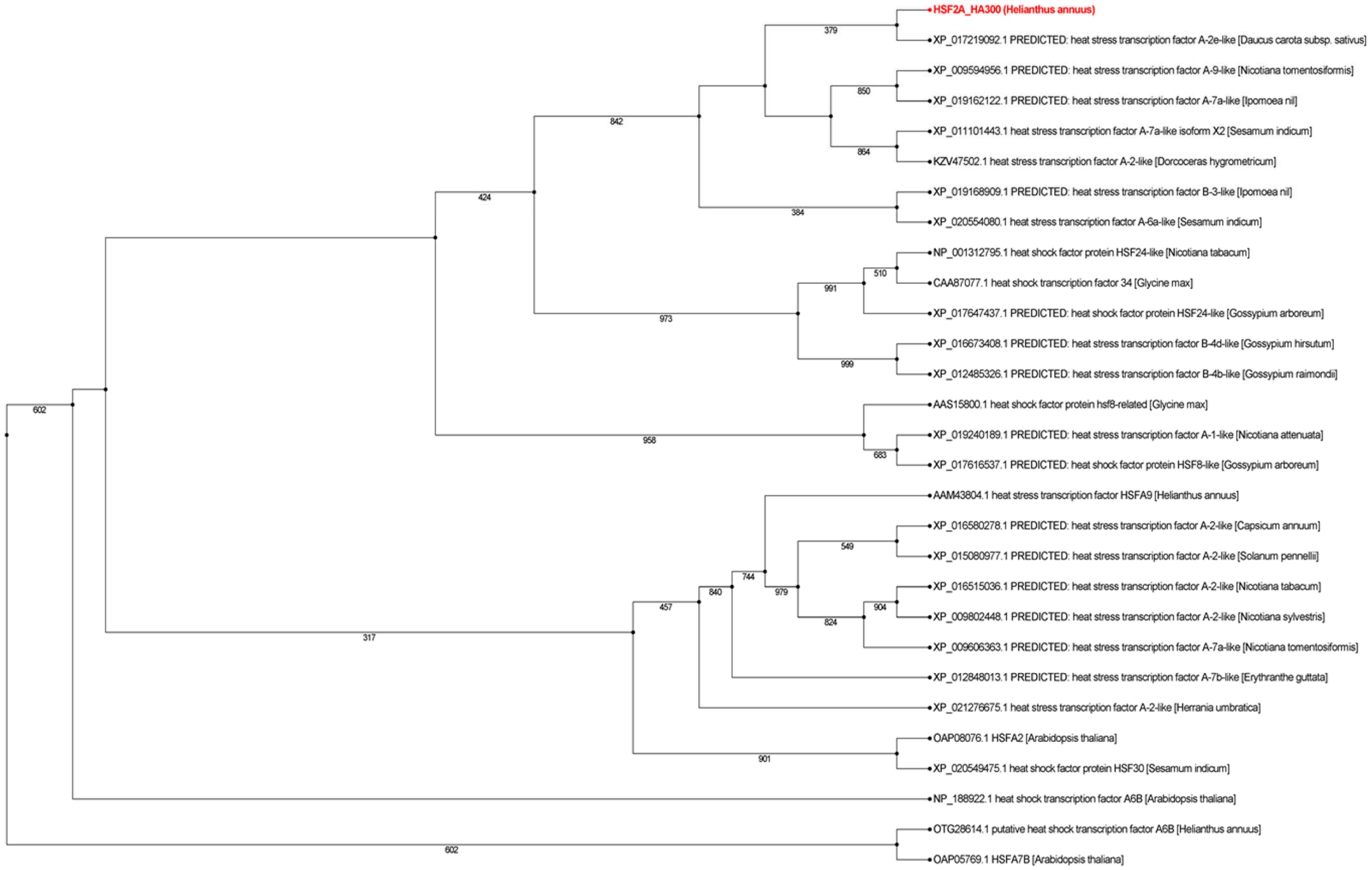

Figure S5. Phylogenetic tree of the *HSF* gene in plants. Nodes are labeled with their bootstrap support values.

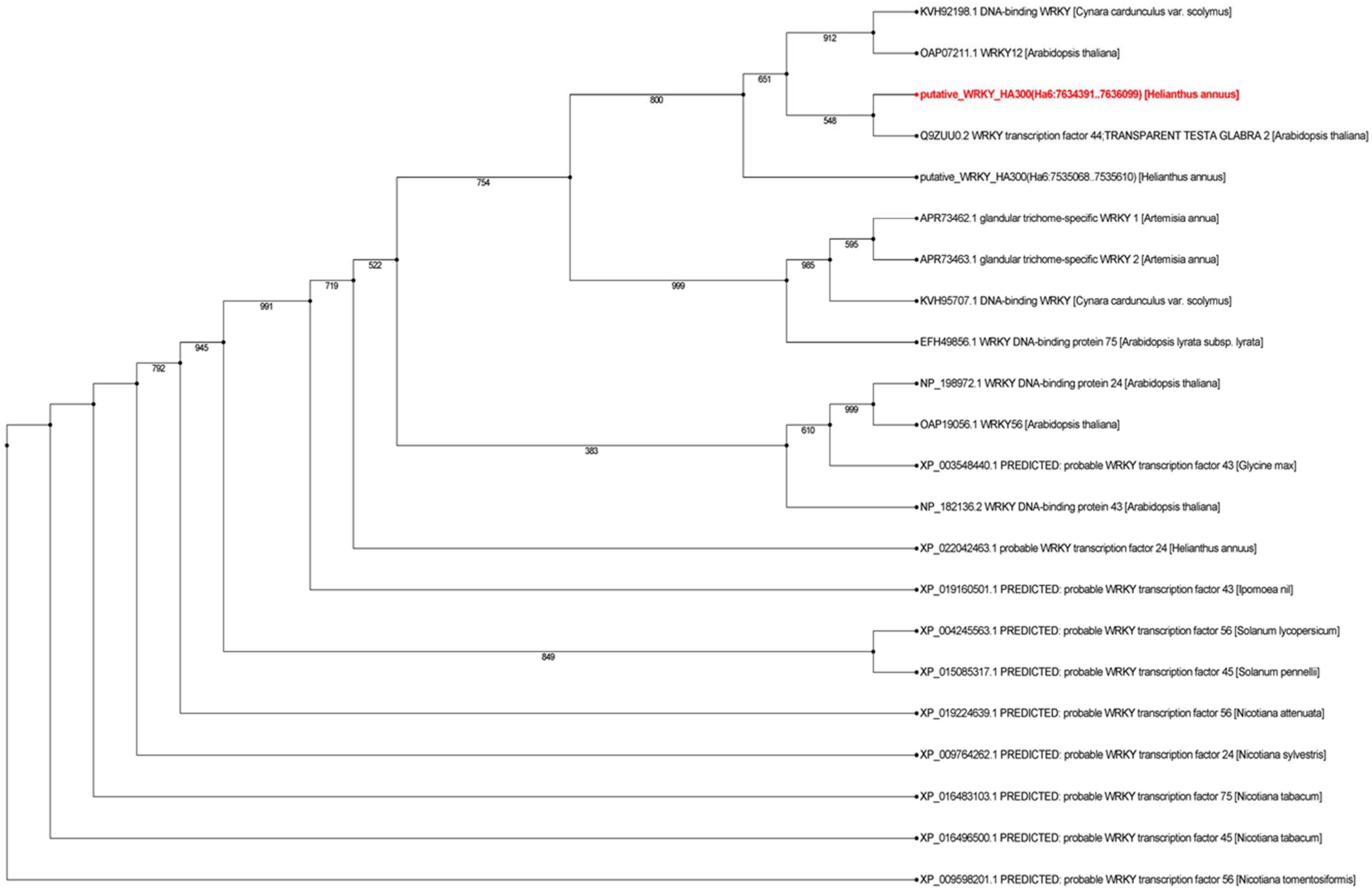

Figure S6. Phylogenetic tree of the *WRKY* gene in plants. Nodes are labeled with their bootstrap support values.
